# Synthesis of Naphthalene-Based Push-Pull Molecules with a Heteroaromatic Electron Acceptor

**DOI:** 10.3390/molecules21030267

**Published:** 2016-03-02

**Authors:** David Šarlah, Amadej Juranovič, Boris Kožar, Luka Rejc, Amalija Golobič, Andrej Petrič

**Affiliations:** 1 Faculty of Chemistry and Chemical Technology, University of Ljubljana, Večna pot 113, 1000 Ljubljana, Slovenia; sarlah@illinois.edu (D.Š.); amadej.juranovic@gmail.com (A.J.); boris.kozar@hotmail.com (B.K.); rejc.luka87@gmail.com (L.R.); amalija.golobic@fkkt.uni-lj.si (A.G.); 2 EN-FIST Centre of Excellence, Trg Osvobodilne fronte 13, SI-1000 Ljubljana, Slovenia

**Keywords:** FDDNP analogs, push-pull dyes, heterocyclization, cross-coupling reactions, UV/vis spectroscopy

## Abstract

Naphthalene derivatives bearing electron-accepting and electron-donating groups at the 2,6-positions belong to the family of D-π-A push-pull dyes. It has been found that these compounds, e.g., 2-(1-(6-((2-(fluoro)ethyl)(methyl)amino)naphthalen-2-yl)ethylidene)malononitrile (FDDNP), show not only interesting optical properties, such as solvatochromism, but they have the potential to label protein aggregates of different compositions formed in the brain of patients suffering from neurodegenerative diseases like Alzheimer’s (AD). In continuation of our research we set our goal to find new FDDNP analogs, which would inherit optical and binding properties but hopefully show better specificity for tau protein aggregates, which are characteristic for neurodegeneration caused by repetitive mild trauma. In this work we report on the synthesis of new FDDNP analogs in which the acceptor group has been formally replaced with an aromatic five- or six-membered heterocycle. The heterocyclic moiety was annealed to the central naphthalene ring either by classical ring closure reactions or by modern transition metal-catalyzed coupling reactions. The chemical characterization, NMR spectra, and UV/vis properties of all new compounds are reported.

## 1. Introduction

**Figure 1:**
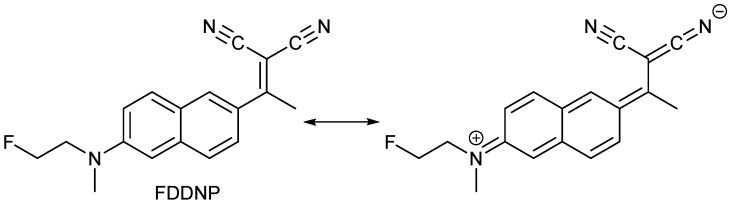
Limiting resonance forms showing electron delocalization in FDDNP.

Molecules with a delocalized π-system end-capped with an electron donor (D) and an acceptor (A) group (D-π-A) are widely utilized in optoelectronic devices and other functional materials. Intense research was dedicated to determine the effects of the donor, acceptor, and the π-system parts of a push-pull chromophore on its properties [1]. The interaction between the donor and the acceptor (intramolecular charge transfer, ICT) is mediated by the π-system; it can be expressed by limiting resonance forms as exemplified for 2-(1-(6-((2-(fluoro)ethyl)(methyl)amino)naphthalen-2-yl)-ethylidene)malononitrile (FDDNP, Figure 1).

The extent of delocalization and consequently the corresponding HOMO-LUMO gap as well as optical properties are governed by several factors:

(i) The donor group. The most efficient donor groups are those exhibiting positive resonance effect (+R). Of those, *N,N*-dialkylamino groups proved to be one of the most efficient and frequently utilized in D-π-A molecules.

The spatial arrangement of the substituents at the donor nitrogen atom was found to influence the donor efficacy. In aziridine and piperidine, the ring is puckered and the substituents around the nitrogen are pushed out of the plane. This pyramidal arrangement hinders an efficient delocalization of the nitrogen lone electron pair towards the A-group. In open-chain dialkylamines, azetidine, or pyrrolidine the arrangement is planar, resulting in an efficient delocalization of the electron pair; this is reflected in absorption maxima at longer wavelengths and smaller chemical shifts of neighboring protons in NMR spectra [2];

(ii) The π-system. As the π-link between the A and D groups in D-π-A molecules usually serves an aromatic/heteroaromatic ring alone or combined with a conjugated alkene or alkyne structural element. It has been shown, that a conjugation with an alkene structural element leads to higher A–D interactions than the acetylene one [1,3].
(iii) The acceptor. Groups with negative resonance (–R) and/or inductive effects (–I), such as cyano, nitro and carbonyl are the most efficient acceptors. As the A group, five or six-membered electron-deficient heterocycles, such as thiazole, benzo[*d*]thiazole, imidazole, pyrazine, pyridine *etc.* bearing one or more electron-withdrawing substituents, can also be applied [1,4].

**Figure 2:**
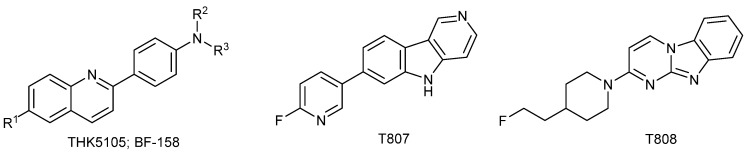
Molecular structures of selected heterocyclic molecular probes.

2-(1-(6-(Dimethylamino)naphthalen-2-yl)ethylidene)malononitrile (DDNP) was initially developed as a solvent polarity/viscosity dependent fluorescent D-π-A type dye, which can be utilized for tissue staining in fluorescence microscopy [5]. We have used its analogue FDDNP with different *in vitro* and *in vivo* techniques to detect changes in the central nervous system present in the brain of the patients suffering from a variety of neurodegenerative diseases [6,7,8]. In conjunction with positron emission tomography (PET), radiolabeled [^18^F]FDDNP was used as the molecular probe for the diagnosis of the Alzheimer’s disease in living patients [9]. In recent years, a number of *in vitro* and *in vivo* imaging techniques for the detection of protein deposits in the CNS has been developed in order to help diagnose the disease in early stages [10]. New compounds derived from fused aromatic heterocycles like THK5105, T807, or T808 (Figure 2), have been reported as imaging agents aimed to protein labeling [11,12,13,14,15,16,17]. Several of them show selectivity for tau protein aggregates over amyloid‒β plaques; this opens a possibility to distinguish between the pathologies.

In contrast to PET and autoradiography, which require radiolabeled tracers, fluorescence microscopy is a more widely accessible *in vitro* method. For this technique, besides the prerequisite specific labeling interaction with the protein aggregates, the molecular probe has to be fluorescent. To diminish the contribution to the background signal resulting from endogenous fluorescent molecules, which are typically excited in the UV range, the preferred excitation maxima of the molecular probe candidates are above 400 nm. The most efficient autofluorescence suppression has been reported for the near-infrared fluorescent probes, which are typically excited around 600 nm and emit in the NIR range [18]. Several attempts were made to identify the molecular components responsible for optical and binding properties of FDDNP and its analogs. It has been found that changes at the donor side of the molecule did not bring about the desired optical properties [19,20,21] as the absorption/excitation maxima wavelengths of the reported derivatives did not exceed those of the lead compound. In contrast, optical properties proved to be much more sensitive to the nature of the acceptor group [2]. The ability of a molecule to attain a planar conformation without a substantial energy input has also been identified as an important governing factor. It affects optical properties and the ability of binding into the hydrophobic channel running the length of the longitudinal fibril axis [2].

The rationale behind the design of a new heteroaromatic moiety containing FDDNP analogs was to offer the means for fine-tuning the electron accepting ability of the group and to introduce nitrogen atoms capable of serving as additional interaction points with a protein. The planned structural modifications feature a single bond between the naphthalene ring and the substituents, facilitating a low-energy rotation of the substituents relative to the central naphthalene ring and preserving the conjugation between the donor and the acceptor sites in the molecule. The electronic effects of the heteroaryl groups on these derivatives can be further varied through a careful selection of the substituents on the heterocyclic ring and/or through a variation of the heteroatom position.

## 2. Results and Discussion

Herein, we describe a preparation of FDDNP analogs in which the acceptor side of the molecule has been either elaborated into or formally replaced by a five- or six-membered heteroaryl group. Both classical and modern approaches from the toolbox of organic synthetic chemistry were chosen to provide an access to the envisioned compounds. The synthesis of 4-naphthylpyridine derivatives from DDNP utilizing a Knoevenagel condensation and heterocyclization steps has been previously reported from this laboratory [4]. In this work, the acetyl group in 1-(6-(dimethylamino)naphthalen-2-yl)ethan-1-one has been transformed into a 1,3-diketo side-chain, which can be further elaborated into a five- or six membered heterocyclic ring with a number of 2- or 3-atom synthons. In addition to this classical approach, modern, selective transition metal-catalyzed C–C bond-forming reactions were envisaged for a direct introduction of a heteroaryl group to the central naphthalene ring. Absorption spectra of all new compounds were recorded in solvents of different polarity to document the effect of the acceptor group modification on optical properties and to serve as a preliminary test of their potential application as the molecular probes with standard fluorescence microscopes. The hydroxyl group at the amine side chains in the compounds **19**–**24** can be utilized as the reactive site for radiolabeling transformations. This opens the possibility of testing their binding to amyloid-β and tau proteins by autoradiography and potential application with PET *in vivo* imaging, irrespective to their applicability with fluorescence microscopy *in vitro*.

**Scheme 1:**
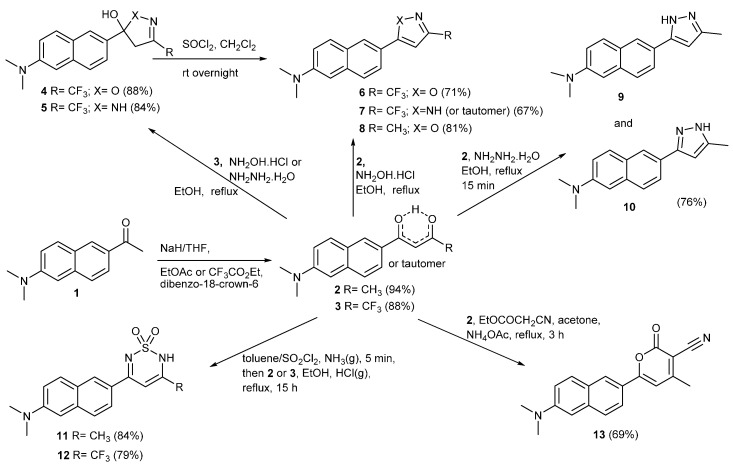
Syntheses of heteroaryl DDNP derivatives by cyclisation reactions.

The 1,3-diketones **2** and **3**, requisite for the heterocyclization step, were prepared via a Claisen condensation reaction of 1-(6-(dimethylamino)naphthalen-2-yl)ethan-1-one (**1**) [5] with ethyl acetate or ethyl trifluoroacetate, respectively (Scheme 1).

Although dubbed as 1,3-diketones, the compounds **2** and **3** exist in solution predominately in their corresponding enol tautomeric forms. This was supported by the presence of two signals in the respective ^1^H-NMR spectra in CDCl_3_. The signals at 6.25 ppm and 6.60 ppm were attributed to the alkene protons of **2** and **3**, respectively; the signals with large chemical shifts (16.39 ppm for **2** and 15.52 ppm for **3**) were assigned to the intramolecular hydrogen-bonded hydroxyl groups. The spectrum of **2** revealed the presence of another set of signals, which were assigned to the keto tautomer. Based on the peak areas the ratio between the enol and the keto tautomer was estimated to be 9:1. In the spectrum of **3** no signals for the keto tautomer were detected; the compound **3** in CDCl_3_ exists exclusively in the enol form. Both compounds were further elaborated into isoxazole, pyrazole, and cyclic sulfonamide derivatives using classical heterocyclization reactions with bidentate nucleophilic reagents as depicted in Scheme 1.

**Figure 3:**
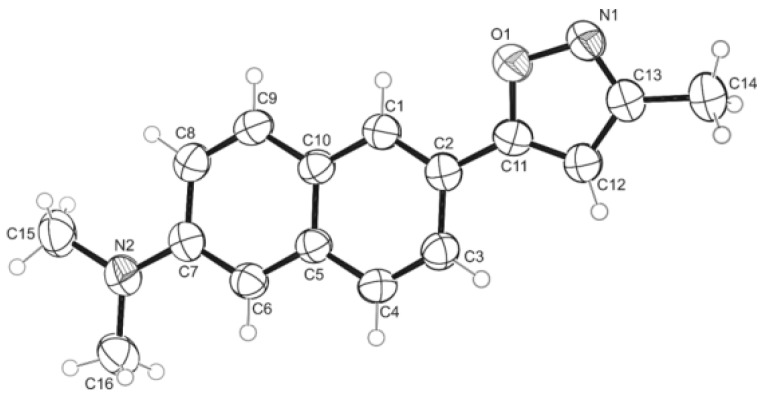
ORTEP drawing of compound **8**. For details please see the Supplementary Materials.

The compound **2** reacted regioselectively with hydroxylamine hydrochloride in boiling EtOH to yield a single product **8**. Based on the NMR spectral data, it was not possible to differentiate between the two possible isomeric structures, namely 3-methylisoxazol-5-yl and 5-methylisoxazol-3-yl. The unequivocal proof of the isoxazole **8** structure was acquired by single crystal x-ray analysis (Figure 3).

Upon treatment with hydrazine monohydrate in refluxing EtOH, the compound **2** underwent heterocyclization yielding a pyrazole derivative. Careful analysis of the NMR spectra revealed the presence of two inseparable tautomers (**9** and **10**) in the ratio of approximately 1:2. In similar reactions of the diketone **3** with hydroxylamine hydrochloride or hydrazine monohydrate, dihydroisoxazole intermediates **4** and **5** were stable enough to be isolated and characterized. The intermediates **4** and **5** were dehydrated into isoxazole derivatives **6** and **7**, respectively, by a treatment with either thionyl chloride followed by the addition of a catalytic amount of pyridine, or NaOH in MeOH at room temperature [22,23].

Sulfurous diamide, freshly prepared from thionyl chloride and gaseous ammonia in toluene, reacted at reflux in EtOH with **2** or **3** to give the cyclic sulfonamides **11** and **12**, respectively. A Knoevenagel-type condensation of the diketone **2** with ethyl cyanoacetate, followed by a ring closure resulted in the pyranone derivative **13**. Analogous reactions with the diketone **3** at different temperatures have led to complex mixtures in which the expected pyranone product could not be detected. Low solubility of **13** in standard NMR solvents hampered the acquisition of ^13^C-NMR spectral data; however, ^1^H-NMR spectroscopy, high-resolution mass spectroscopy, infrared spectroscopy, and the melting point measurement confirmed the identity and purity of the product.

**Scheme 2:**
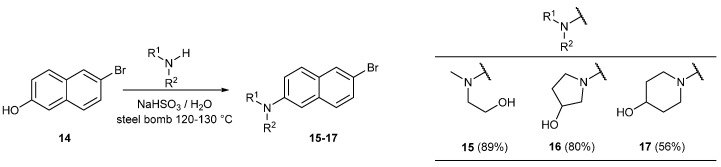
Syntheses of 2-bromonaphth-6-ylamines.

Modern cross-coupling reactions provide an efficient and regioselective approach to C–C bond formation. These transformations also tolerate the presence of a wide variety of functional groups and solvents [24,25]. A Suzuki-type cross-coupling between a heteroaromatic boronic acid and an aryl halide was chosen as a synthetic route to the desired heteroaryl-substituted naphthylamines. To this end, 6-bromonaphthalen-2-ol (**14**) was first transformed with secondary amines by Bucherer reactions into 6-bromonaphth-2-ylamines **15**, **16**, and **17** (Scheme 2) [2].

**Scheme 3:**
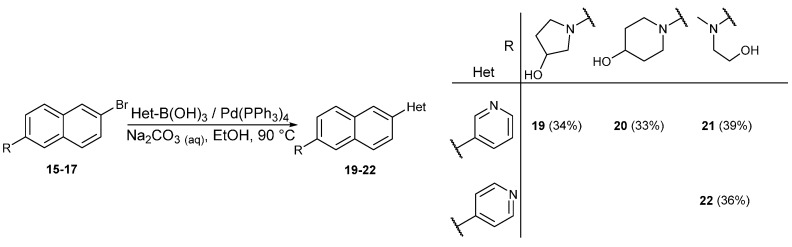
Syntheses of heteroaryl DDNP analogs by the Suzuki coupling using heteroarylboronic acids.

The subsequent palladium-catalyzed Suzuki cross-coupling reactions with commercially available pyridylboronic acids were carried out under biphasic conditions in an organic solvent and with aqueous sodium carbonate to give the pyridyl derivatives **19**–**22** in relatively poor yields (Scheme 3). The yields of these compounds were inferior to those reported for the dimethylamino analogues **24**–**26** described below. We have attributed these differences in yields to a problematic separation of the products **19**–**22** from tarry side-products of similar polarity. A free hydroxyl group in the D-group in these compounds makes them much more polar in comparison to the dimethylamino group-containing compounds **24**–**26**.

**Scheme 4:**
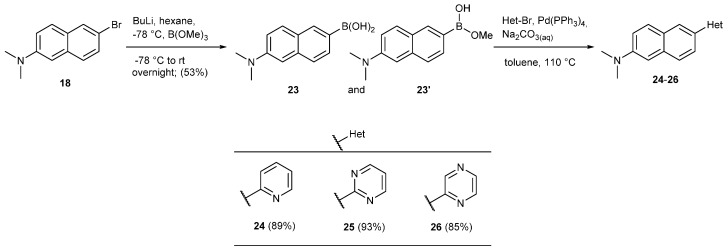
Syntheses of heteroaryl DDNP analogs by the Suzuki coupling.

An absence of protic sites in 6-bromo-*N,N*-dimethylnaphthalen-2-amine (**18**) enabled an alternative synthetic pathway (Scheme 4). The compound **18** was first subjected to a transmetalation reaction with *tert*-butyl lithium. The resulting organolithium compound was transformed into an intermediary boronic acid ester, which was then hydrolyzed into the boronic acid **23** [26]. However, under the employed reaction conditions the hydrolysis was not complete, and a 1:1 mixture of the acid **23** and its monomethyl ester **23**′ was isolated (Scheme 4).

Nevertheless, the mixture of **23** and **23**′ was successfully used in a subsequent Suzuki cross-coupling reaction under reaction conditions similar to those described above. The intended products **24**, **25**, and **26** were formed in 89%, 93%, and 85% yields, respectively; this suggested that both, the boronic acid **23** and its monomethyl ester **23**′ participated in the reaction.

**Scheme 5:**
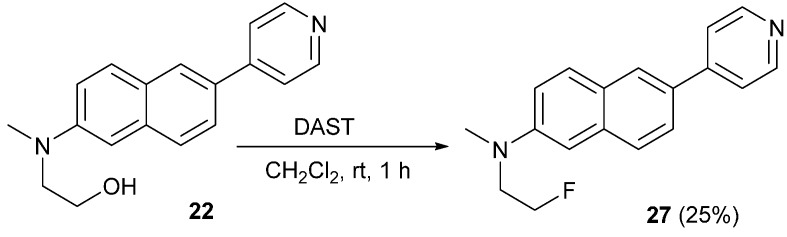
Synthesis of the fluoro compound **27**.

Out of four compounds **15**–**17** and **22**, containing the side-chain with a free hydroxyl group, the latter was selected to test the synthetic procedure for hydroxy-to-fluoro group exchange. A treatment with diethylaminosulfur trifluoride (DAST) yielded fluoro derivative **27** (Scheme 5); this transformation provided a compound, which formally differs from FDDNP only at the acceptor side of the molecule. Thus, the differences in optical properties between FDDNP and **27** can be attributed mainly to the difference in the electron-attracting character of the different acceptor groups.

Similar to what we have found previously with other FDDNP derivatives, the variation at the electron donor site in the above new compounds exerted only a minor effect on the optical properties [2]. The effect of the changes at the electron acceptor site was much more pronounced. Absorption maxima of the compounds with pyrazole, oxazole, pyridine, pyrimidine, or pyrazine rings as electron-accepting group are in the range between 320 and 345 nm, showing only slight dependence on solvent polarity (Table 1). This indicates that the above-mentioned heterocyclic rings are weaker electron acceptors than the 1,1-dicyanopropenyl group in FDDNP/DDNP compound family thus making these derivatives less suitable molecular probes for their application in fluorescence microscopy. On the other hand, the cyclic sulfonamides **11** and **12** and the pyranone carbonitrile **13** are all visibly fluorescent and exhibit similar absorption maxima to those measured for FDDNP/DDNP in a hydrophobic environment. These compounds have the potential to be applied as fluorescent probes in fluorescence imaging.

However, our goal is to find new molecular probes, which would be applied *in vivo* imaging with PET. Because the optical properties are not a limiting factor for PET, the binding properties to amyloid-β and tau protein aggregates of all new compounds remain to be determined in competitive binding assays *in vitro* using [^18^F]FDDNP.

## 3. Materials and Methods

### 3.1. General Information

Radial chromatography was performed using a Chromatotron model 7924T (Harrison Research, Palo Alto, CA, USA). Rotors were coated with silicagel 60 PF_254_ containing gypsum for preparative layer chromatography in 1, 2, or 4 mm thick layers. Silicagel 130–270 Mesh 60 A was used for column chromatography. Melting points were determined in an Optimelt MPA 100 instrument, (Stanford Research Systems, Sunnyvale, CA, USA). NMR spectra of CDCl_3_ or DMSO-*d*_6_ solutions were recorded on Avance DPX 300 MHz (Bruker, Rheinstetten Germany) at 302 K; chemical shifts are reported relative to tetramethylsilane (TMS) or residual solvent peaks. Elemental analyses were performed on a 2400 CHNS/O (Perkin Elmer, Waltham, MA, USA). HRMS spectra were obtained on an 6224 Accurate Mass TOF LC/MS instrument (Agilent, Santa Clara, CA, USA).

### 3.2. Synthesis of 1-(6-(Dimethylamino)naphthalen-2-yl)butan-1,3-dione *(**2**)*

To a suspension of NaH (1.12 g, 46.7 mmol) in THF (40 mL) EtOAc (4.6 mL, 46.8 mmol) was added under argon and stirred for 30 min. A solution of 1-(6-(dimethylamino)naphthalen-2-yl)ethan-1-one [5] (**1**; 2 g, 9.4 mmol) and dibenzo-18-crown-6 (50 mg, 0.14 mmol) in THF (40 mL) was added to the reaction mixture and stirred at rt for 30 min followed by heating at reflux temperature for 5 h. The solvent was removed by rotary evaporation and the resulting mixture suspended in the mixture of EtOAc and concentrated aqueous solution of NH_4_Cl. The suspension was acidified with aqueous solution of HCl (1 M) to the pH 3–4 and the product was extracted from the water phase with EtOAc. The combined organic phases were treated with NaHCO_3_, dried over anhydrous sodium sulfate, and the solvent was removed under reduced pressure. The crude product was crystallized from EtOH to yield **2** (2.24 g, 8.75 mmol, 94%; enol:keto = 9:1) as pale yellow crystals; m.p. 148.8 °C–151.9 °C. ^1^H-NMR (300 MHz, CDCl_3_): δ = 2.19 (s, 3H), 3.08 (s, 6H), 6.25 (s, 1H), 6.93 (d, ^4^*J*_H,H_ = 2.4 Hz, 1H), 7.14 (dd, ^3^*J*_H,H_ = 9.1 Hz, ^4^*J*_H,H_ = 2.4 Hz, 1H), 7.62 (d, ^3^*J*_H,H_ = 8.7 Hz, 1H) 7.7–7.81 (m, 2H), 8.29 (d, ^4^*J*_H,H_ = 1.9 Hz, 1H), 16.39 (bs, 1H). ^13^C-NMR (75 MHz, CDCl_3_): δ = 25.5, 40.4, 96.1, 105.3, 116.3, 123.7, 125.4, 126.2, 127.8, 128.3, 130.4, 137.3, 150.0, 184.2, 191.9. HRMS *m*/*z* [M + H]^+^ calcd for C_16_H_18_NO_2_: 256.1332; found 256.1340. Anal. calcd for C_16_H_17_NO_2_ (%): C: 75.27; H: 6.71; N: 5.49; found: C, 75.05; H: 6.79; N: 5.50.

### 3.3. Synthesis of 1-(6-(Dimethylamino)naphthalen-2-yl)-4,4,4-trifluorobutan-1,3-dione *(**3**)*

To a suspension of NaH (2.24 g, 93.3 mmol) in THF (50 mL) a solution of CF_3_CO_2_Et (5.6 mL, 66.8 mmol), **1** (4 g, 18.8 mmol), and dibenzo-18-crown-6 (250 mg, 0.7 mmol) in THF (70 mL) were slowly added under argon atmosphere and stirred for 30 min. Stirring was continued at reflux temperature overnight. Upon cooling, the solvent was removed by rotary evaporation and the resulting mixture was suspended in the mixture of EtOAc and concentrated aqueous solution of NH_4_Cl. The suspension was acidified with aqueous solution of HCl (1 M) to pH 3–4 and the product was extracted with EtOAc. The combined organic phases were washed with NaHCO_3_, dried over anhydrous sodium sulfate, and the solvent was removed under reduced pressure. The crude product was crystallized from EtOH to yield **3** (5.11 g, 16.48 mmol, 88%; enol tautomer) as red-orange crystals; m.p. 98.8 °C–101.1 °C. ^1^H-NMR (300 MHz, CDCl_3_): δ = 3.10 (s, 6H), 6.60 (s, 1H), 6.81 (d, ^4^*J*_H,H_ = 2.5 Hz, 1H), 7.13 (dd, ^4^*J*_H,H_ = 2.5 Hz, ^3^*J*_H,H_ = 9.1 Hz, 1H), 7.60 (d, ^3^*J*_H,H_ = 8.8 Hz, 1H), 7.74 (d, ^3^*J*_H,H_ = 9.1 Hz, 1H), 7.76 (dd, ^3^*J*_H,H_ = 8.8 Hz, ^4^*J*_H,H_ = 1.8 Hz, 1H), 8.23 (d, ^4^*J*_H,H_ = 1.8 Hz, 1H), 15.52 (bs, 1H). ^13^C-NMR (75 MHz, CDCl_3_): δ = 40.22, 91.59 (q, *J* = 2.1 Hz), 105.06, 116.35, 117.52 (q, *J* = 283.0 Hz), 123.44, 125.01, 125.29, 126.49, 129.95, 131.00, 138.35, 150.69, 175.75 (q, *J* = 35.73 Hz), 186.41. HRMS *m*/*z* [M + H]^+^ calcd for C_16_H_15_F_3_NO_2_: 310.1049; found 310.1054. Anal. calcd for C_16_H_14_F_3_NO_2_ (%): C, 62.13; H, 4.56; N, 4.53; found C, 62.55; H, 4.68; N, 4.50.

### 3.4. Synthesis of 5-(6-(Dimethylamino)naphthalen-2-yl)-3-(trifluoromethyl)-4,5-dihydroisoxazol-5-ol *(**4**)*

To a solution of the diketone **3** (618 mg, 2.0 mmol) in EtOH (20 mL), NH_2_OH × HCl (152.5 mg, 2.2 mmol) was added and the reaction mixture was heated at reflux for 4 h. The solvent was then removed under reduced pressure; the residue was dissolved in EtOAc and washed with NaHCO_3_. The organic phase was dried and the solvent was removed by rotary evaporation. The crude product was purified by crystallization from EtOH to give **4** (570 mg, 1.76 mmol, 88%) as light yellow crystals; m.p. 202 °C–203 °C. ^1^H-NMR (300 MHz, DMSO-*d*_6_): δ = 3.05 (s, 6H), 3.62 (d, ^2^*J*_H,H_ = 18.3 Hz, 1H), 3.97 (d, ^2^*J*_H,H_ = 18.3 Hz, 1H), 6.96 (d, ^4^*J*_H,H_ = 2.5 Hz, 1H), 7.27 (dd, ^3^*J*_H,H_ = 9.1 Hz, ^4^*J*_H,H_ = 2.5 Hz, 1H), 7.67–7.71 (m, 2H), 7.79 (d, ^3^*J*_H,H_ = 9.1 Hz, 1H), 7.99 (s, 1H), 8.77 (s, 1H). ^13^C-NMR (75 MHz, DMSO-*d*_6_): δ = 39.9, 42.3, 103.2 (q, ^2^*J*_C,F_ = 32.9 Hz), 105.2, 116.5, 120.8, 122.5 (q, ^1^*J*_C,F_ = 284.6 Hz), 122.9, 125.0, 126.4, 127.6, 129.3, 135.6, 149.3, 157.1. HRMS *m*/*z* [M + H]^+^ calcd for C_16_H_16_F_3_N_2_O_2_: 325.1159; found: 325.1158.

### 3.5. Synthesis of N,N-Dimethyl-6-(3-(trifluoromethyl)isoxazol-5-yl)naphthalen-2-amine *(**6**)*

To a solution of **4** (1.04 g, 3.2 mmol) in CH_2_Cl_2_ (150 mL), thionyl chloride (0.49 mL, 6.37 mmol) was added at room temperature and the solution was stirred overnight. Pyridine (3 drops) was added to the solution and the reaction mixture heated at reflux for 5 h. The solvent was removed under reduced pressure, the residue was suspended in a mixture of EtOAc and concentrated aqueous solution of NaHCO_3_. The water phase was extracted with three portions of EtOAc, 80 mL each. The combined organic phases were dried, and the solvent was removed by rotary evaporation. The crude product was purified by crystallization from EtOH to yield **6** as a white crystalline powder (695 mg, 2.27 mmol, 71%); m.p. 149.8 °C–150.9 °C. ^1^H-NMR (300 MHz, DMSO-*d*_6_): δ = 3.05 (s, 6H), 6.98 (d, ^4^*J*_H,H_ = 2.3 Hz, 1H), 7.29 (dd, ^4^*J*_H,H_ = 2.3 Hz, ^3^*J*_H,H_ = 9.1 Hz, 1H), 7.71 (d, ^3^*J*_H,H_ = 8.7 Hz, 1H), 7.82 (d, ^3^*J*_H,H_ = 9.1 Hz, 1H) 7.87 (dd, ^3^*J*_H,H_ = 8.7 Hz, ^4^*J*_H,H_ = 1.5 Hz, 1H), 8.08 (s, 1H), 8.35 (d, ^4^*J*_H,H_ = 1.5 Hz, 1H). ^13^C-NMR (75 MHz, DMSO-*d*_6_): δ = 39.89, 105.1, 105.2 (q, ^3^*J*_C,F_ = 2.2 Hz), 116.7, 117.9 (q, ^1^*J*_C,F_ = 268.7 Hz), 119.3, 123.4, 125.2, 126.8, 127.07, 129.3, 135.8, 149.4, 156.9 (q, ^2^*J*_C,F_ = 41.6), 162.9. HRMS *m*/*z* [M]^+^ calcd for C_16_H_13_F_3_N_2_O: 306.0978; found: 306.0972.

### 3.6. Synthesis of 5-(6-(Dimethylamino)naphthalen-2-yl)-3-(trifluoromethyl)-4,5-dihydro-1Hpyrazol-5-ol *(**5**)* and N,N-dimethyl-6-(3-(trifluoromethyl)-1H-pyrazol-5-yl)naphthalen-2-amine *(**7**)*

A solution of the diketone **3** (309 mg, 1.0 mmol) in EtOH (20 mL) was heated to 60 °C and hydrazine monohydrate (55 mg, 1.1 mmol) was added. The mixture was heated at reflux for 15 min. Upon cooling, EtOH was removed under reduced pressure. The crude product was purified by crystallization from EtOH/CHCl_3_ (5:1) to yield a pure **5** (271 mg, 0.84 mmol, 84%); m.p. 235 °C–237 °C. HRMS *m*/*z* [M + H]^+^ calcd for C_16_H_17_F_3_N_3_O: 324.1318; found: 324.1319.

To the solution of the dihydropirazole **5** (100 mg, 0.31 mmol) in MeOH (10 mL), NaOH (24.8 mg, 0.62 mmol) was added. After stirring the reaction mixture for 3 days the solvent was removed under reduced pressure. The product was extracted with boiling toluene and the solvent removed by rotary evaporation. The crude material was crystallized from toluene to yield **7** as yellow crystals (63 mg, 0.21 mmol, 67%); m.p. 202 °C–203 °C. ^1^H-NMR (300 MHz, DMSO-*d*_6_): δ = 3.01 (s, 6H), 6.93 (d, ^4^*J*_H,H_ = 2.4 Hz, 1H), 7.03 (s, 1H), 7.22 (dd, ^3^*J*_H,H_ = 9.1 Hz, ^4^*J*_H,H_ = 2.4 Hz, 1H), 7.67–7.72 (m, 2H), 7.83 (dd, ^3^*J*_H,H_ = 8.6 Hz, ^4^*J*_H,H_ = 1.7 Hz, 1H), 8.19 (d, ^4^*J*_H,H_ = 1.7 Hz, 1H). ^13^C-NMR (75 MHz, DMSO-*d*_6_): δ = 41.1, 99.4 (q, ^3^*J*_C,F_ = 1.7 Hz), 105.5, 116.62, 122.7 (q, ^1^*J*_C,F_ = 269.0 Hz), 123.2, 123.9, 124.0, 126.0, 126.2, 128.6, 134.0, 141.2 (q, ^2^*J*_C,F_ = 35.3 Hz) 146.4, 148.4. HRMS *m*/*z* [M]^+^ calcd for C_16_H_14_F_3_N_3_: 305.1140; found: 305.1146.

### 3.7. Synthesis of N,N-Dimethyl-6-(3-methylisoxazol-5-yl)naphthalen-2-amine *(**8**)*

To a solution of the diketone **2** (511 mg, 2.0 mmol) in EtOH (20 mL), NH_2_OH × HCl (152.5 mg, 2.2 mmol) was added and the reaction mixture was heated at reflux for 4 h. The solvent was then removed under reduced pressure; the residue was dissolved in EtOAc and washed with NaHCO_3_. The organic phase was dried and the solvent was removed by rotary evaporation. The crude product was purified by crystallization from EtOH to give **8** as white needles (408 mg, 1.62 mmol, 81%); m.p. 98.8 °C–101.1 °C. ^1^H-NMR (300 MHz, CDCl_3_): δ = 2.34 (s, 3H), 3.05 (s, 6H), 6.31 (s, 1H), 6.85 (d, ^4^*J*_H,H_ = 2.5 Hz, 1H), 7.14 (dd, ^3^*J*_H,H_ = 9.1 Hz, ^4^*J*_H,H_ = 2.5 Hz, 1H), 7.64 (m, 2H), 7.72 (d, ^3^*J*_H,H_ = 9.1 Hz, 1H), 8.09 (s, 1H). ^13^C-NMR (75 MHz, CDCl_3_): δ = 11.5, 40.5, 99.1, 105.7, 116.6, 120.8, 123.3, 125.1, 125.9, 126.7, 129.5, 135.6, 149.3, 160.2, 170.3. HRMS *m*/*z* [M + H]^+^ calculated for C_16_H_17_N_2_O: 253.1335; found 253.1340. Anal. calcd for C_16_H_16_N_2_O (%): C, 76.16; H, 6.39; N, 11.10; found C, 76.68; H, 6.48; N, 11.13.

### 3.8. Synthesis of N,N-Dimethyl-6-(3-methyl-1H-pyrazol-5-yl)naphthalen-2-amine *(**9**)* and N,N-dimethyl-6-(5-methyl-1H-pyrazol-3-yl)naphthalen-2-amine *(**10**)*

A solution of the diketone **2** (255 mg, 1.0 mmol) in EtOH (20 mL) was heated to 60 °C and hydrazine monohydrate (55 mg, 1.1 mmol) was added. The mixture was heated at reflux for 15 min. Upon cooling, EtOH was removed under reduced pressure. The residue was purified by crystallization from a mixture of EtOH/toluene (1:1) to give a mixture of **9** and **10** as bright brown crystals (190 mg, 0.76 mmol, 76%); m.p. 198.3 °C–199.1 °C. ^1^H-NMR (300 MHz, DMSO-*d*_6_): δ = 2.27 (s, 3H), 3.00 (s, 6H), 6.46 (s, 1H), 6.93 (d, ^4^*J*_H,H_ = 2.5 Hz, 1H), 7.22 (dd, ^3^*J*_H,H_ = 9.1 Hz, ^4^*J*_H,H_ = 2.5 Hz, 1H), 7.60–7.90 (m, 3H), 8.04 (bs, 1H), 12.47 (s, 0.6H, NH), 12.80 (s, 0.4H, NH). ^13^C-NMR (75 MHz, DMSO-*d*_6_): δ = 11.7, 40.2, 100.8, 105.6, 116.5, 122.8, 123.8, 126.1, 126.1, 128.5, 133.9, 139.5, 142.9, 148.3, 150.8 (note: chemical shifts of signals at 139.5, 142.9, and 150.8 were determined from HMBC and HMQC NMR spectra). HRMS *m*/*z* [M + H]^+^ calculated for C_16_H_18_N_3_: 252.1495; found: 252.1499.

### 3.9. General Procedure for the Synthesis of Cyclic Sulfonamides

Into a solution of SO_2_Cl_2_ (1 mL) in toluene (50 mL) at 0 °C, gaseous NH_3_ was bubbled in for 3 to 5 min. The mixture was evaporated and the solution of the corresponding diketone (2.0 mmol) in EtOH (30 mL), saturated with gaseous HCl was added to the resulting white solid. The suspension was heated at reflux for 15 h. The solvent was removed by rotary evaporation and the resulting residue was suspended in a mixture of water and EtOH (1:1). The precipitated product was filtered-off and crystallized.

#### 3.9.1. *N*,*N*-Dimethyl-6-(3-methyl-1,1-dioxido-2*H*-1,2,6-thiadiazin-5-yl)naphthalen-2-amine (**11**)

Crystallized from EtOH/H_2_O (3:1) as light yellow crystals (529 mg, 1.68 mmol, 84%); m.p. 260 °C–262 °C. ^1^H-NMR (300 MHz, DMSO-*d*_6_): δ = 2.27 (s, 3H), 3.09 (s, 6H), 6.73 (s, 1H), 7.24 (b, 1H), 7.40 (dd, ^3^*J*_H,H_ = 9.1 Hz, ^4^*J*_H,H_ = 2.4 Hz, 1H), 7.79 (d, ^3^*J*_H,H_ = 8.8 Hz, 1H), 7.90–8.00 (m, 2H), 8.53 (d, ^4^*J*_H,H_ = 1.7 Hz, 1H). ^13^C-NMR (75 MHz, DMSO-*d*_6_): δ = 20.63, 40.9, 95.9, 107.7, 117.2, 124.3, 126.3, 126.7, 128.2, 128.8, 130.5, 136.2, 148.4, 159.4, 165.4. HRMS *m*/*z* [M + H]^+^ calculated for C_16_H_18_N_3_O_2_S: 316.1114; found: 316.1114.

#### 3.9.2. Synthesis of 6-[1,1-Dioxido-3-(trifluoromethyl)-2*H*-1,2,6-thiadiazin-5-yl]-*N*,*N*-dimethyl-naphthalen-2-amine (**12**)

Crystallized from EtOH/Me_2_CO (4:1) as yellow-red crystals (583 mg, 1.58 mmol, 79%); m.p. 245 °C–248 °C. ^1^H-NMR (300 MHz, DMSO-*d*_6_): δ = 3.21 (s, 6H), 6.60 (s, 1H), 7.60 (dd, ^4^*J*_H,H_ = 2.4 Hz, ^3^*J*_H,H_ = 9.1 Hz, 1H), 7.75 (d, ^4^*J*_H,H_ = 2.4 Hz, 1H), 7.93 (d, ^3^*J*_H,H_ = 8.7 Hz, 1H), 8.08 (dd, ^3^*J*_H,H_ = 8.7 Hz, ^4^*J*_H,H_ = 1.7 Hz, 1H), 8.13 (d, ^3^*J*_H,H_ = 9.1 Hz, 1H), 8.56 (bd, ^4^*J*_H,H_ = 1.7 Hz, 1H), 9.35 (bs, 1H, NH). ^13^C-NMR (75 MHz, DMSO-*d*_6_): δ = 43.6, 88.7, 113.7 (q, ^3^*J*_C,F_ = 1.9 Hz), 118.0, 120.8 (q, ^1^*J*_C,F_ = 277.6 Hz), 124.9, 127.1, 127.6, 129.5, 131.0, 132.7, 134.97, 144.5, 154.1 (q, ^2^*J*_C,F_ = 33.3 Hz), 163.5. HRMS *m*/*z* [M + H]^+^ calculated for C_16_H_15_F_3_N_3_O_2_S: 370.0832; found: 370.0838.

### 3.10. Synthesis of 6-[6-(Dimethylamino)naphthalen-2-yl]-4-methyl-2-oxo-2H-pyran-3-carbonitrile *(**13**)*

To a solution of the diketone **2** (511 mg, 2.0 mmol) in acetone (15 mL), EtOCOCH_2_CN (1 mL) and NH_4_OAc (2 g) were added. The reaction mixture was heated at reflux for 3 h. Upon cooling, the precipitate was removed by vacuum filtration, washed with water and EtOH, and air dried. The crude product was purified by crystallization from CH_2_Cl_2_/hexane to give **13** as orange-red crystals (420 mg, 1.38 mmol, 69%); m.p. 288 °C–290 °C. ^1^H-NMR (300 MHz, DMSO-*d*_6_): δ = 2.47 (s, 3H), 3.09 (s, 6H), 6.89 (d, ^4^*J*_H,H_ = 2.5 Hz, 1H), 7.30 (dd, ^3^*J*_H,H_ = 9.1 Hz, ^4^*J*_H,H_ = 2.5 Hz, 1H), 7.38 (s, 1H), 7.76 (d, ^3^*J*_H,H_ = 8.8 Hz, 1H), 7.84 (dd, ^4^*J*_H,H_ = 1.9 Hz, ^3^*J*_H,H_ = 8.8 Hz, 1H), 7.92 (d, *J* = 9.1 Hz, 1H), 8.40 (d, ^4^*J*_H,H_ = 1.9 Hz, 1H). HRMS [M + H]^+^ *m*/*z* calculated for C_19_H_17_N_2_O_2_: 305.1284; found: 305.1292.

### 3.11. General Procedure for the Bucherer Reaction

A steel bomb was charged with 6-bromo-2-naphthol (**14**), a secondary amine, sodium bisulfite, and water. The mixture was stirred at 120 °C–130 °C for 72 h. Upon cooling the reaction mixture was diluted with water, the product was extracted with CH_2_Cl_2_ or EtOAc, and the organic layer was washed with 5% NaOH solution, followed by washing with brine. The organic phase was dried over anhydrous sodium sulfate and the solvent was removed by rotary evaporation. The crude product was purified by crystallization to give the corresponding naphthylamine.

#### 3.11.1. Synthesis of 2-((6-Bromonaphthalen-2-yl)(methyl)amino)ethan-1-ol (**15**)

A mixture of 6-bromo-2-naphthol (**14**) (5.15 g, 18.4 mmol), 2-(methylamino)ethanol (15.8 mL, 22.7 g, 0.303 mol), NaHSO_3_ (11 g, 0.106 mol), and water (17 mL) was treated as described in the general procedure for the Bucherer reaction; the product was extracted with EtOAc and purified by crystallization from MeOH/H_2_O to give **15** as a grey amorphous solid (4.57 g, 16.38 mmol, 89%); m.p. 81.5 °C–82.5 °C. ^1^H-NMR (300 MHz, CDCl_3_): δ = 3.04 (s, 3H), 3.55 (t, ^3^*J*_H,H_ = 5.6 Hz, 2H), 3.86 (bt, ^3^*J*_H,H_ = 5.5 Hz, 2H), 6.91 (d, ^4^*J*_H,H_ = 2.5 Hz, 1H), 7.19 (dd, ^3^*J*_H,H_ = 9.1 Hz, ^4^*J*_H,H_ = 2.5 Hz, 1H), 7.41 (dd, ^3^*J*_H,H_ = 8.8 Hz, ^4^*J*_H,H_ = 1.9 Hz, 1H), 7.50 (d, ^3^*J*_H,H_ = 8.8 Hz, 1H), 7.59 (d, ^3^*J*_H,H_ = 9.1 Hz, 1H), 7.81 (d, ^4^*J*_H,H_ = 1.9 Hz, 1H). ^13^C-NMR (75 MHz, CDCl_3_): δ = 39.1, 55.6, 60.4, 106.8, 115.6, 117.6, 128.1, 128.2, 128.3, 129.57, 129.7, 133.6, 148.38. HRMS *m*/*z* [M]^+^ calculated for C_13_H_14_BrNO: 279.0259; found 279.0261. Anal. calcd for C_13_H_14_BrNO (%): C, 55.73; H, 5.04; N, 5.00; found: C, 56.05; H, 4.94; N, 5.04.

#### 3.11.2. Synthesis of 1-(6-Bromonaphthalen-2-yl)pyrrolidin-3-ol (**16**)

A mixture of 6-bromo-2-naphthol (**14**) (2.1 g, 9.4 mmol), 3-hydroxypyrrolidine (5 g, 57.4 mmol), NaHSO_3_ (8 g, 76.9 mmol), and water (25 mL) was treated as described in the general procedure for the Bucherer reaction; the product was extracted with EtOAc and purified by radial chromatography (silica gel, EtOAc/hexane = 1/2) followed by crystallization from MeOH/H_2_O to give **16** (2.23 g, 7.66 mmol, 80%); m.p. 163.9 °C–165.5 °C. ^1^H-NMR (300 MHz, CDCl_3_): δ = 1.66 (d, ^3^*J*_H,H_ = 4.7 Hz, 1H), 2.04–2.30 (m, 2H), 3.37–3.66 (m, 4H), 4.62–4.70 (m, 1H), 6.72 (d, ^4^*J*_H,H_ = 2.2 Hz, 1H), 6.99 (dd, ^3^*J*_H,H_ = 9.0 Hz, ^4^*J*_H,H_ = 2.4 Hz, 1H), 7.40 (dd, ^3^*J*_H,H_ = 8.8 Hz, ^4^*J*_H,H_ = 2.0 Hz, 1H), 7.50 (d, ^3^*J*_H,H_ = 8.8 Hz, 1H), 7.60 (d, ^3^*J*_H,H_ = 9.0 Hz, 1H), 7.82 (d, ^4^*J*_H,H_ = 1.9 Hz, 1H). ^13^C-NMR (75 MHz, DMSO-*d_6_*): δ = 33.7, 45.5, 56.0, 69.3, 103.8, 112.9, 116.6, 126.8, 127.6, 127.9, 128.7, 129.1, 133.4, 145.9. HRMS *m*/*z* [M]^+^ calculated for C_14_H_14_BrNO: 291.0259; found 291.0261. Anal. calcd for C_14_H_14_BrNO (%): C, 57.55; H, 4.83; N, 4.79; found: C, 57.43; H, 4.60; N, 4.72.

#### 3.11.3. Synthesis of 1-(6-Bromonaphthalen-2-yl)piperidin-4-ol (**17**)

A mixture of 6-bromo-2-naphthol (**14**) (2 g, 9 mmol), 4-hydroxypiperidine (3 g, 29.6 mmol), NaHSO_3_ (6 g, 57.7 mmol), and water (15 mL) was treated as described in the general procedure for the Bucherer reaction; the product was extracted with CH_2_Cl_2_, and purified by crystallization from EtOH to give **17** as white crystals (1.54 g, 5.05 mmol, 56%): m.p. 135.8 °C–138.8 °C. ^1^H-NMR (300 MHz, CDCl_3_): δ = 1.68–1.79 (m, 2H), 2.01–2.09 (m, 2H), 2.98–3.06 (m, 2H), 3.62–3.69 (m, 2H), 3.86–3.94 (m, 1H), 7.07 (d, ^4^*J*_H,H_ = 2.5 Hz, 1H), 7.28 (dd, ^3^*J*_H,H_ = 9.1 Hz, ^4^*J*_H,H_ = 2.5 Hz, 1H), 7.44 (dd, ^3^*J*_H,H_ = 8.7 Hz, ^4^*J*_H,H_ = 2.0 Hz, 1H), 7.53 (d, ^3^*J*_H,H_ = 8.8 Hz, 1H), 7.61 (d, ^3^*J*_H,H_ = 9.1 Hz, 1H), 7.84 (d, ^4^*J*_H,H_ = 2.0 Hz, 1H). ^13^C-NMR (75 MHz, CDCl_3_): δ = 43.3, 47.4, 67.9, 110.3, 116.7, 120.8, 128.0, 128.5, 129.4, 129.5, 129.6, 133.3, 149.5. HRMS *m*/*z* [M]^+^ calculated for C_15_H_16_BrNO: 305.0415; found 305.0421. Anal. calcd for C_15_H_16_BrNO (%): C, 58.84; H, 5.27; N, 4.57; found: C, 58.62; H, 5.27; N 4.60.

### 3.12. General Procedure for the Suzuki Coupling

**Procedure A:** A glass vial was charged with aryl bromide and purged with argon. A solution of tetrakistriphenylphosphine palladium(0) in dimethoxyethane and an aqueous solution of NaHCO_3_ (2 M) were added. The solution was stirred at room temperature for 5 min when aryl boronic acid in 2 mL EtOH was added. The reaction mixture was stirred at 90 °C until TLC revealed that the product content was not increasing any more. Upon cooling the reaction mixture was diluted with water and the product extracted with CH_2_Cl_2_. The organic layers were combined, dried over anhydrous sodium sulfate, and the solvent was removed. The crude product was isolated by radial chromatography (silica gel) and purified by crystallization.

**Procedure B:** A solution of 2-bromo-6-dimethylaminonaphthalene (**18**; 8.32 g, 33.3 mmol) [2] in THF (100 mL) was cooled to −78 °C before BuLi in hexane (2.7 M, 12.3 mL, 33.3 mmol) was added. After stirring for 1 h at −78 °C, trimethoxy borate (3.7 mL, 33.3 mmol) was added slowly, the reaction mixture was let to warm up to the ambient temperature and stirred overnight. The reaction was quenched by an addition of concentrated aqueous solution of ammonium chloride (50 mL). After washing the water phase several times with diethyl ether, the organic phases were combined, washed with 0.5 M solution of HCl, followed by washing with 5% NaHCO_3_. Upon drying, the solvent was removed and the residue washed with hexane to give a mixture of the boronic acid **23** [26] and its monomethyl ester (3.8 g, 53%); m.p. > 300 °C. HRMS *m*/*z* [M + H]^+^ calculated for C_12_H_15_BNO_2_: 216.1190; found 216.1194. HRMS *m*/*z* [M + H]^+^ calculated for monomethyl ester C_13_H_16_BNO_2_: 230.1347; found 230.1352.

A vial was charged with an aqueous solution of sodium carbonate (1.1 mL, 2 M, 2.2 mmol), toluene (7 mL), 6-(dimethylamino)naphthyl-2-boronic acid (**23**; 215 mg, 1.0 mmol) and halogenated heteroaromatic compound (1.0 mmol). The reaction mixture was purged with argon, sealed and heated overnight at 110 °C with stirring. Upon cooling, the product was extracted with EtOAc, the organic phase was washed with an aqueous solution of NaHCO_3_, dried over anhydrous sodium sulfate, and the solvent was removed under vacuum. The crude product was purified by column chromatography, followed by crystallization.

#### 3.12.1. Synthesis of 1-(6-(Pyridin-3-yl)naphthalen-2-yl)pyrrolidin-3-ol (**19**)

Arylbromide **16** (292 mg, 1 mmol), Pd(PPh_3_)_4_ (45 mg, 0.039 mmol), Na_2_CO_3_ in water (2 M, 1.5 mL), and 3-pyridinyl boronic acid (136 mg, 1.1 mmol) in EtOH (5 mL) were treated as described in the procedure A for 2.5 h; the product was extracted with hot *i*-PrOH, the precipitate formed after cooling was filtered-off, and the remaining solid crystallized from MeOH to give a pure solid **19** (98 mg, 0.34 mmol, 34%); m.p. 237 °C–239 °C. ^1^H-NMR (300 MHz, DMSO-*d_6_*): δ = 1.91–2.16 (m, 2H), 3.21–3.57 (m, 4H), 4.43–4.49 (m, 1H), 5.00 (d, ^4^*J*_H,H_ = 3.8 Hz, 1H), 6.77 (d, ^4^*J*_H,H_ = 2.1 Hz, 1H), 7.06 (dd, ^3^*J*_H,H_ = 9.0 Hz, ^3^*J*_H,H_ = 2.3 Hz, 1H), 7.47 (dd, ^3^*J*_H,H_ = 8.0 and 4.7 Hz, 1H), 7.70 (dd, ^3^*J*_H,H_ = 8.6 Hz, ^4^*J*_H,H_ = 1.7 Hz, 1H), 7.75 (d, ^3^*J*_H,H_ = 8.7 Hz, 1H), 7.82 (d, ^3^*J*_H,H_ = 9.0 Hz, 1H), 8.08 (d, ^4^*J*_H,H_ = 1.5 Hz, 1H), 8.14 (ddd, *J* = 8.0, 1.5, and 1.5 Hz, 1H), 8.53 (dd, *J* = 4.8 and 1.5 Hz, 1H), 8.98 (d, ^4^*J*_H,H_ = 2.1 Hz, 1H). HRMS *m*/*z* [M]^+^ calculated for C_19_H_18_N_2_O: 290.1419; found 290.1425. Anal. calcd for C_19_H_18_N_2_O (%): C, 78.59; H, 6.25; N, 9.65; found C, 78.33; H, 6.31; N, 9.65.

#### 3.12.2. Synthesis of 1-(6-(Pyridin-3-yl)naphthalen-2-yl)piperidin-4-ol (**20**)

Arylbromide **17** (306 mg, 1 mmol), Pd(PPh_3_)_4_ (45 mg, 0.039 mmol), Na_2_CO_3_ in water (2 M, 1.5 mL), and 3-pyridinyl boronic acid (139 mg, 1.1 mmol) in EtOH (2 mL) were treated as described in the procedure A for 2 h; the product was purified twice by crystallization from hot *i*-PrOH to give a pure **20** (100 mg, 0.33 mmol, 33%); m.p. 212 °C–214 °C. ^1^H-NMR (300 MHz, DMSO-*d*_6_): δ = 1.47–1.59 (m, 2H), 1.84–1.92 (m, 2H), 2.94–3.03 (m, 2H), 3.64–3.73 (m, 3H), 4.70 (d, ^3^*J*_H,H_ = 4.2 Hz, 1H), 7.21 (d, ^4^*J*_H,H_ = 2.4 Hz, 1H), 7.41 (dd, ^3^*J*_H,H_ = 9.1 Hz, ^4^*J*_H,H_ = 2.5 Hz, 1H), 7.49 (dd, ^3^*J*_H,H_ = 8.0 and 4.7 Hz, 1H), 7.75 (d, ^3^*J*_H,H_ = 8.6 Hz, ^4^*J*_H,H_ = 1.9 Hz, 1H), 7.79–7.85 (m, 2H), 8.12 (d, ^4^*J*_H,H_ = 1.7 Hz, 1H), 8.16 (ddd, *J* = 8.0, 1.6, and 1.6 Hz, 1H), 8.55 (dd, *J* = 4.7 and 1.4 Hz, 1H), 9.00 (d, ^4^*J*_H,H_ = 2.1 Hz, 1H). ^13^C-NMR (75 MHz, DMSO-*d*_6_): δ = 33.8, 46.3, 66.0, 108.5, 119.6, 123.2, 124.7, 125.2, 127.3, 127.4, 128.9, 130.9, 133.7, 134.1, 135.7, 147.5, 147.9, 149.2. HRMS *m*/*z* [M]^+^calculated for C_20_H_20_N_2_O: 304.1576; found 304.1582. Anal. calcd for C_20_H_20_N_2_O (%): C, 78.92; H, 6.62; N, 9.20; found C, 78.74; H, 6.88; N, 9.12.

#### 3.12.3. Synthesis of 2-(Methyl(6-(pyridin-3-yl)naphthalen-2-yl)amino)ethan-1-ol (**21**)

Arylbromide **15** (140 mg, 0.5 mmol), Pd(PPh_3_)_4_ (30 mg, 0.029 mmol), Na_2_CO_3_ in water (2 M, 0.5 mL), and 3-pyridinyl boronic acid (70 mg, 0.63 mmol) in EtOH (2 mL) were treated as described in the procedure A for 1 h; the product was purified by radial chromatography (silica gel, EtOAc: CH_2_Cl_2_ = 20:1) and crystallized from hot acetonitrile to give a pale yellow crystalline solid **21** (54 mg, 0.19 mmol, 39%); m.p. 154.0 °C–156.5 °C. ^1^H-NMR (300 MHz, CDCl_3_): δ = 2.57 (b, 1H), 3.08 (s, 3H), 3.61 (t, ^3^*J*_H,H_ = 5.5 Hz, 2H), 3.89 (t, ^3^*J*_H,H_ = 5.6 Hz, 2H), 6.94 (d, ^4^*J*_H,H_ = 2.4 Hz, 1H), 7.20 (dd, ^3^*J*_H,H_ = ^3^*J*_H,H_ = 9.1 Hz, 2.4 Hz, 1H), 7.35 (dd, ^3^*J*_H,H_ = 7.8 Hz, ^4^*J*_H,H_ = 4.7 Hz, 1H), 7.51 (dd, ^3^*J*_H,H_ = 8.7 Hz, ^4^*J*_H,H_ = 1.8 Hz, 1H), 7.66 (d, ^3^*J*_H,H_ = 8.7 Hz, 1H), 7.70 (d, ^3^*J*_H,H_ = 8.7 Hz, 1H), 7.81 (d, ^4^*J*_H,H_ = 1.8 Hz, 1H), 7.94 (dt, ^3^*J*_H,H_ = 7.8 Hz, ^4^*J*_H,H_ = 2.0 Hz, 1H), 8.53 (dd, ^3^*J*_H,H_ = 5.3 Hz, ^4^*J*_H,H_ = 1.6 Hz, 1H), 8.84 (d, ^4^*J*_H,H_ = 2.0 Hz, 1H). ^13^C-NMR (75 MHz, CDCl_3_): δ = 39.1, 55.6, 60.3, 106.2, 117.2, 123.8, 125.4, 125.9, 127.1, 127.3, 129.5, 131.2, 134.4, 134.8, 137.2, 147.8, 148.2, 148.5. Anal. calcd for C_18_H_18_N_2_O (%): C, 77.67; H, 6.52; N, 10.06; found C, 77.63; H, 6.54; N, 10.01.

#### 3.12.4. Synthesis of 2-(Methyl(6-(pyridin-4-yl)naphthalen-2-yl)amino)ethan-1-ol (**22**)

Arylbromide **15** (420 mg, 1.5 mmol), Pd(PPh_3_)_4_ (60 mg, 0.058 mmol), Na_2_CO_3_ in water (2 M, 1.5 mL), and 4-pyridinyl boronic acid (230 mg, 1.7 mmol, 90%) in EtOH (2 mL) were treated as described in the procedure A for 2 h; the product was purified by radial chromatography (silica gel, EtOAc: CH_2_Cl_2_ = 6:1) and crystallized from hot acetonitrile to give a pale yellow crystalline solid **22** (150 mg, 0.54 mmol, 36%); m.p. 207.5 °C–208.5 °C. ^1^H-NMR (300 MHz, CDCl_3_): δ = 3.12 (s, 3H), 3.54 (t, ^3^*J*_H,H_ = 5.6 Hz, 2H), 3.61 (t, ^3^*J*_H,H_ = 5.6 Hz, 2H), 4,72 (t, ^3^*J*_H,H_ = 5.3 Hz, 1H), 6.94 (d, ^4^*J*_H,H_ = 2.4 Hz, 1H), 7.25 (dd, ^3^*J*_H,H_ = 9.1 Hz, ^4^*J*_H,H_ = 2.4 Hz, 1H), 7.70–7.85 (m, 5H), 8.20 (s, 1H), 8.61 (m, 2H). ^13^C-NMR (75 MHz, CDCl_3_): δ = 36.1, 59.9, 64.0, 110.0, 122.1, 126.3, 129.9, 131.4, 131.4, 134.9, 135.0, 135.0, 140.9, 152.8, 153.7, 155.8. Anal. calcd for C_18_H_18_N_2_O (%): C, 77.67; H, 6.52, N, 10.06; found C, 77.92; H, 6.79; N, 10.20.

#### 3.12.5. Synthesis of *N*,*N*-Dimethyl-6-(pyridin-2-yl)naphthalen-2-amine (**24**)

The crude product was obtained according to the procedure B; the product was purified by column chromatography (silica gel, CH_2_Cl_2_/PE = 3/1) and crystallized from EtOH to give a pure **24** (221 mg, 0.89 mmol, 89%); m.p. 113.1 °C–114.7 °C. ^1^H-NMR (300 MHz, CDCl_3_): δ = 3.05 (s, 6H), 6.92 (d, ^4^*J*_H,H_ = 2.6 Hz, 1H), 7.14–7.19 (m, 2H), 7.68–7.83 (m, 4H), 8.03 (dd, ^3^*J*_H,H_ = 8.6 Hz, ^4^*J*_H,H_ = 1.9 Hz, 1H), 8.35 (d, ^4^*J*_H,H_ = 1.8 Hz, 1H), 8.70 (ddd, *J* = 4.8, 1.7, and 0.9 Hz, 1H). ^13^C-NMR (75 MHz, CDCl_3_): δ = 40.7, 105.9, 116.5, 120.1, 121.4, 124.8, 126.0, 126.6, 126.6, 129.6, 132.8, 135.3, 136.6, 149.0, 149.6, 157.7. HRMS *m*/*z* [M + H]^+^ calculated for C_17_H_17_N_2_: 249.1396; found 249.1395. Anal. calcd for C_17_H_16_N_2_ (%): C, 82.22; H, 6.49; N, 11.28; found C, 82.42; H, 6.77; N, 10.91.

#### 3.12.6. Synthesis of *N*,*N*-Dimethyl-6-(pyrimidin-2-yl)naphthalen-2-amine (**25**)

The crude product was obtained according to the procedure B; the product was purified by column chromatography (silica gel, CH_2_Cl_2_:PE = 3:2) and crystallized from toluene to give a pure **25** (232 mg, 0.93 mmol, 93%); m.p. 199 °C–200 °C. ^1^H-NMR (300 MHz, CDCl_3_): δ = 3.08 (s, 6H), 6.93 (d, ^4^*J*_H,H_ = 2.5 Hz, 1H), 7.12 (t, ^3^*J*_H,H_ = 4.8 Hz, 1H), 7.17 (dd, ^3^*J*_H,H_ = 9.1 Hz, ^4^*J*_H,H_ = 2.5 Hz, 1H), 7.73 (d, ^3^*J*_H,H_ = 8.7 Hz, 1H), 7.84 (d, ^3^*J*_H,H_ = 8.9 Hz, 1H), 8.42 (dd, ^3^*J*_H,H_ = 8.7 Hz, ^4^*J*_H,H_ = 1.8 Hz, 1H), 8.78–8.83 (m, 3H). ^13^C-NMR (75 MHz, CDCl_3_): δ = 40.6, 105.8, 116.3, 118.3, 125.4, 126.7, 126.4, 128.3, 130.2, 131.0, 136.5, 149.4, 157.1, 165.2. HRMS *m*/*z* [M + H]^+^ calculated for C_16_H_16_N_3_: 250.1339; found 250.1343.

#### 3.12.7. Synthesis of *N*,*N*-Dimethyl-6-(pyrazin-2-yl)naphthalen-2-amine (**26**)

The crude product was obtained according to the procedure B; the product was purified by column chromatography (silica gel, CH_2_Cl_2_:petroleum ether = 3:1) and crystallized from toluene to give a pure **26** (212 mg, 0.85 mmol, 85%); m.p. 139.7 °C–141.8 °C. ^1^H-NMR (300 MHz, CDCl_3_): δ = 3.08 (s, 6H), 6.92 (d, ^4^*J*_H,H_ = 2.4 Hz, 1H), 7.18 (dd, ^3^*J*_H,H_ = 9.1 Hz, ^4^*J*_H,H_ = 2.4 Hz, 1H), 7.75 (d, ^3^*J*_H,H_ = 8.7 Hz, 1H), 7.81 (d, ^3^*J*_H,H_ = 9.1 Hz, 1H), 8.03 (dd, ^3^*J*_H,H_ = 8.7 Hz, ^4^*J*_H,H_ = 1.8 Hz, 1H), 8.37 (bs, 1H), 8.44 (d, ^3^*J*_H,H_ = 2.5 Hz, 1H), 8.61 (dd, *J* = 2.5 and 1.3 Hz, 1H), 9.12 (d, ^4^*J*_H,H_ = 1.3 Hz, 1H). ^13^C-NMR (75 MHz, CDCl_3_): δ = 40.6, 105.7, 116.6, 124.2, 126.4, 126.5, 127.0, 129.5, 129.8, 135.8, 142.0, 142.0, 144.0, 149.4, 153.2. HRMS *m*/*z* [M + H]^+^ calculated for C_16_H_16_N_3_: 250.1339; found 250.1342.

### 3.13. Synthesis of N-(2-Fluoroethyl)-N-methyl-6-(pyridin-4-yl)naphthalen-2-amine *(**27**)*

To a solution of naphthylamine **22** (50 mg, 0.18 mmol) in CH_2_Cl_2_ in a round bottom flask, sealed with a septum, a solution of DAST (61 mg, 50 μL, 0.38 mmol) in CH_2_Cl_2_ was added drop wise via a syringe. After 1 h, the reaction mixture was poured on ice, the resulting mixture neutralized with NaHCO_3_, and the product was extracted with CH_2_Cl_2_. The organic layers were dried over anhydrous sodium sulfate and the solvent evaporated. The product was purified by radial chromatography (silica gel, 1% MeOH in CH_2_Cl_2_ used as an eluent) and crystallized from hexane/CH_2_Cl_2_ mixture to obtain pure **27** (12.6 mg, 0.05 mmol, 25%) as orange crystals; m.p. 154 °C–157 °C. ^1^H-NMR (300 MHz, CDCl_3_): δ = 3.16 (s, 3H), 3.81 (dt, ^3^*J*_F,H_ = 24.0 Hz, ^3^*J*_H,H_ = 5.1 Hz, 2H), 4.67 (dt, ^2^*J*_F,H_ = 46.5 Hz, ^3^*J*_H,H_ = 5.1 Hz, 2H), 6.94 (d, ^4^*J*_H,H_ = 2.6 Hz, 1H), 7.18 (dd, ^3^*J*_H,H_ = 8.9 Hz, ^4^*J*_H,H_ = 2.6 Hz, 1H), 7.62 (m, 2H), 7.67 (dd, ^3^*J*_H,H_ = 8.6 Hz, ^4^*J*_H,H_ = 1.8 Hz, 1H), 7.75 (d, ^3^*J*_H,H_ = 8.6 Hz, 1H), 7.80 (d, ^3^*J*_H,H_ = 8.9 Hz, 1H), 7.98 (d, ^4^*J*_H,H_ = 1.5 Hz, 1H), 8.65 (bd, *J* = 5,2 Hz). ^13^C-NMR (75 MHz, CDCl_3_): δ = 39.2, 52.8 (d, ^2^*J*_C,F_ = 21.1 Hz), 81.9 (d, ^1^*J*_C,F_ = 170.6 Hz), 105.9, 116.4, 121.4, 124.9, 126.1, 126.7, 127.1, 129.7, 131.3, 135.3, 147.7, 148.7, 150.0. HRMS *m*/*z* [M]^+^calculated for C_18_H_17_FN_2_: 280.1382; found 280.1376. Anal. calcd for C_18_H_17_FN_2_×¼ H_2_O (%): C, 75.90; H, 6.19; N, 9.83; found C, 75.94; H, 6.24; N, 9.65.

### 3.14. Single Crystal X-ray Structure Analysis for Compound ***8***

Single crystal diffraction data for compound **8** have been collected on a Nonius Kappa CCD diffractometer at room temperature with MoK_α_ radiation (0.71073 Å) and graphite monochromator using the Nonius Collect Software [27]. The data were processed using DENZO software [28]. Structure was solved with direct methods, using SIR97 [29]. A full-matrix least-squares refinement on *F^2^* was employed with anisotropic displacement parameters for all non-hydrogen atoms. H atoms were placed at calculated positions and treated as riding; *SHELXL97* software [30] was used for structure refinement and interpretation. Drawing of the structures was produced using *ORTEPIII* [31]. Details of the crystal data, data collection, and refinement parameters have also been deposited with the Cambridge Crystallographic Data Centre as supplementary publication number CCDC 1042435. A copy of the data can be obtained, free of charge, by applying to CCDC, 12 Union Road, Cambridge CB2 1EZ, UK (Fax: +44(0)-1223-336033 or E-Mail: deposit@ccdc.cam.ac.uk).

## 4. Conclusions

A set of new heteroaromatic ring-containing FDDNP/DDNP analogs was synthesized. By a classical heterocyclization approach, 2-pyrazinyl, 5-isoxazolyl, 5-pyrazolyl, 1,2,6-thiadiazin-5-yl, or 2-oxo-2*H*-pyran-6-yl substituent bearing analogs were prepared. Transition metal-catalyzed coupling reactions were used to prepare 2-pyridyl, 3-pyridyl, 4-pyridyl, and 2-pyrimidinyl derivatives. The structures of all new compounds were supported by elemental or HRMS analyses and NMR spectra (^1^H and ^13^C). Based on their UV/vis absorption maxima it was concluded that the cyclic bis-sulfonamides **11** and **12** as well as the 2-oxo-2*H*-pyran-6-yl derivative **13** are the most promising candidates to be applied as dyes in fluorescence microscopy. Other synthesized derivatives exhibit absorption maxima wavelengths similar to those at which also endogenous fluorescent compounds in brain tissue absorb thus rendering them less suitable for the application. However, the ability of the compounds **19**–**22** to be radiolabeled at the side-chain makes them viable candidates for the use as probes in autoradiography *in vitro* or PET imaging *in vivo*, where the appropriate optical properties are not the prerequisite. The effect of structural changes introduced in these compounds on binding to amyloid-β or tau protein and thus their applicability in neurodegeneration imaging remain to be determined in the future.

## Figures and Tables

**Table 1:** Selected UV/vis maxima and molar absorption coefficients of FDDNP, DDNP, and analogs.

	λ_ab_ (nm)					ε × 10^−4^ (L·mol^−1^·cm^−1^)				
	C_6_H_14_	CH_2_Cl_2_	DMF	MeOH	H_2_O	C_6_H_14_	CH_2_Cl_2_	DMF	MeOH	H_2_O
DDNP ^a^	412	438	-	426	-	2.5	2.0	-	2.0	-
FDDNP ^b^	-	431	-	-	-	-	-	-	-	-
**2**	377	394	-	392	371	2.9	2.8	-	3.1	1.3
**3**	384	401	-	384	388	2.0	2.0	-	1.9	1.2
**4**	334	345	-	339	336	2.2	2.3	-	2.2	1.1
**5**	333	339	-	334	328	2.8	2.7	-	2.7	1.5
**6**	329	345	-	339	340	5.9	2.4	-	1.7	0.4
**7**	320	327	-	324	324	1.9	2.0	-	1.9	0.7
**8**	336	342	-	339	330	2.5	2.4	-	2.1	0.6
**9/10**	315	320	-	311	308	2.3	2.3	-	2.4	1.5
**11**	399	425	-	375	364	2.0	2.2	-	2.2	1.6
**12**	412	454	-	389	383	2.1	2.5	-	2.5	1.9
**13**	441	473	-	462	-	2.8	4.7	-	4.3	-
**19**	-	-	335	-	-	-	-	4.5	-	-
**20**	-	-	319	-	-	-	-	6.7	-	-
**24**	334	339	-	337	325	2.4	2.3	-	2.1	1.2
**25**	350	354	-	352	339	2.6	2.2	-	2.1	1.3
**26**	354	363	-	363	343	2.5	2.6	-	2.0	1.6
**27**	328 ^c^	339	342	346	-	2.0 ^c^	1.8	1.9	1.8	-

^a^ from [5]; ^b^ from ref. 2; ^c^ 1% CH_2_Cl_2_ in hexane was used as a solvent.

## Supplementary Materials

The following are available online at http://www.mdpi.com/1420-3049/21/3/267/s1, ^1^H- and ^13^C-NMR spectra of the compounds **2**–**4**, **6**–**13**, **15**–**17**, **19**–**22**, and **24**–**27**.
