# Supplementary material for: Synthesis of Naphthalene-Based Push-Pull Molecules with a Heteroaromatic Electron Acceptor
Source: Molecules. 2016 Mar 2;21(3):267. doi: 10.3390/molecules21030267 (PMC6274339; doi:10.3390/molecules21030267)
Supplement: Supplementary file 1 [file molecules-21-00267-s001.pdf]

Supplementary Material: Synthesis of Naphthalene-Based Push-Pull Molecules with a Heteroaromatic Electron Acceptor

David Šarlah, Amadej Juranovič, Boris Kožar, Luka Rejc, Amalija Golobič and Andrej Petrič

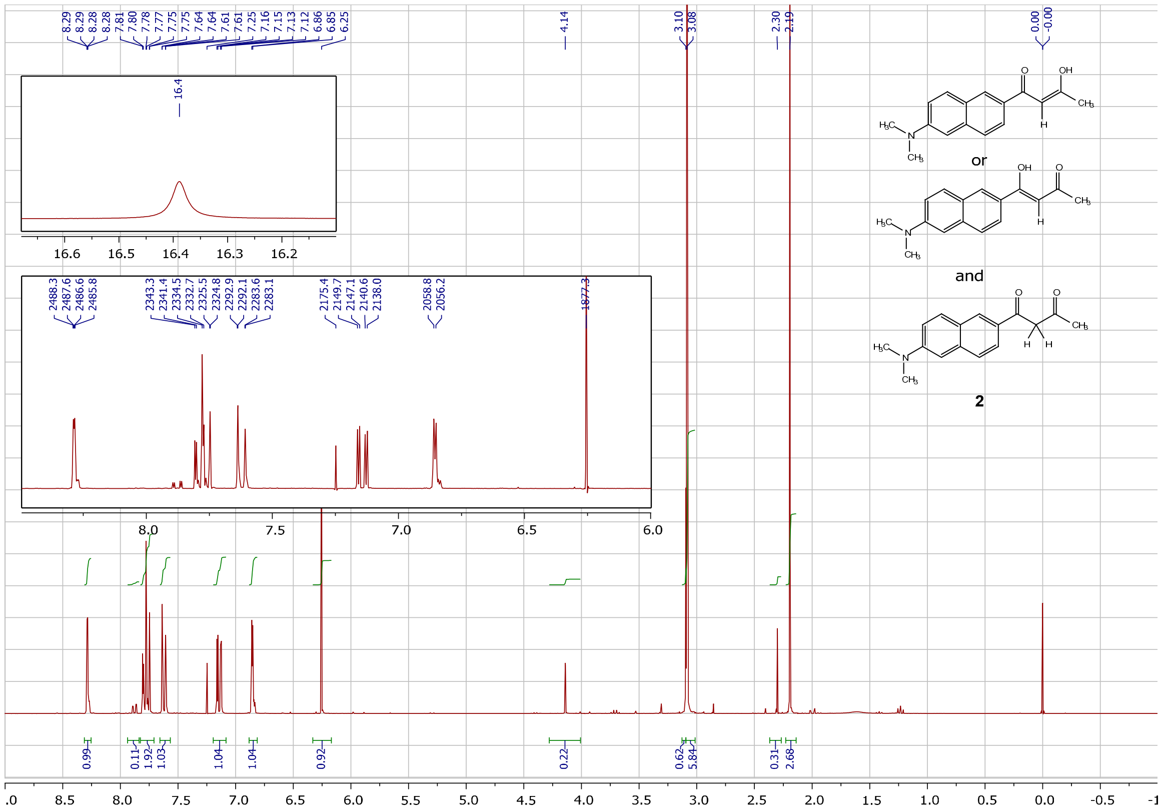

Figure S1. ^{1}H-NMR spectrum of the compound **2**.

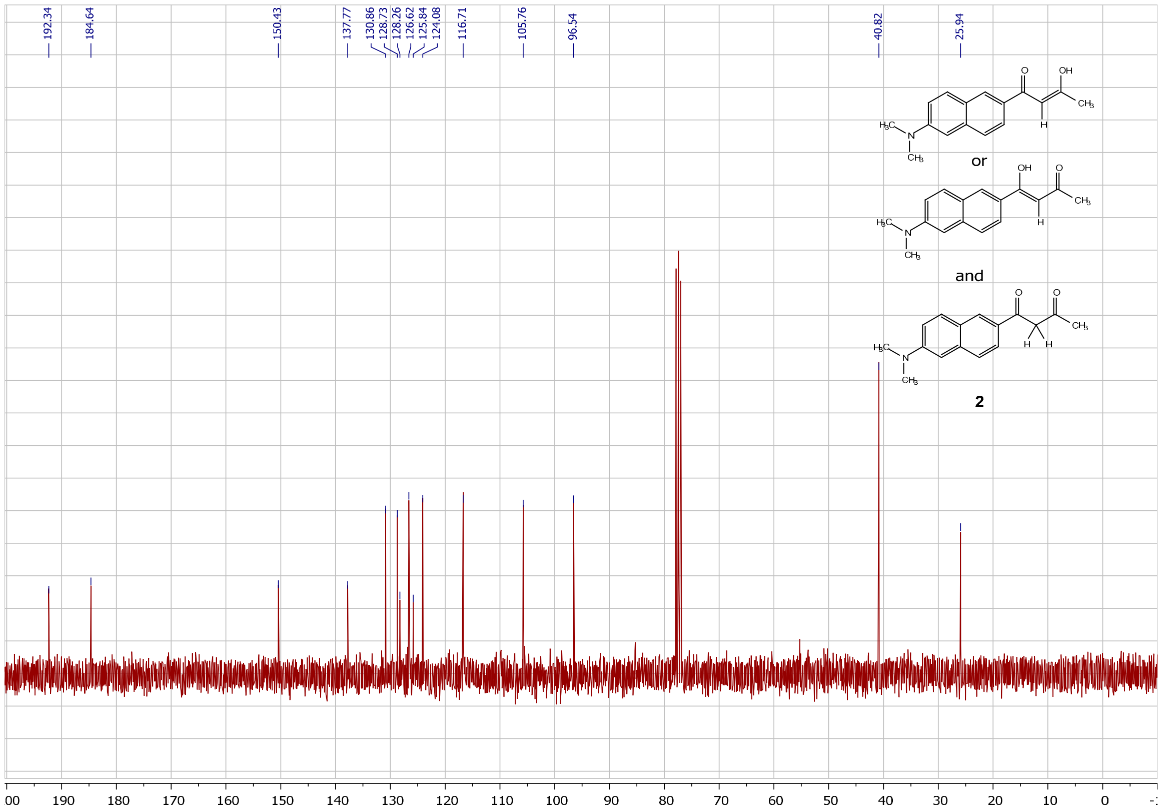

Figure S2. ^{13}C-NMR spectrum of the compound **2**.

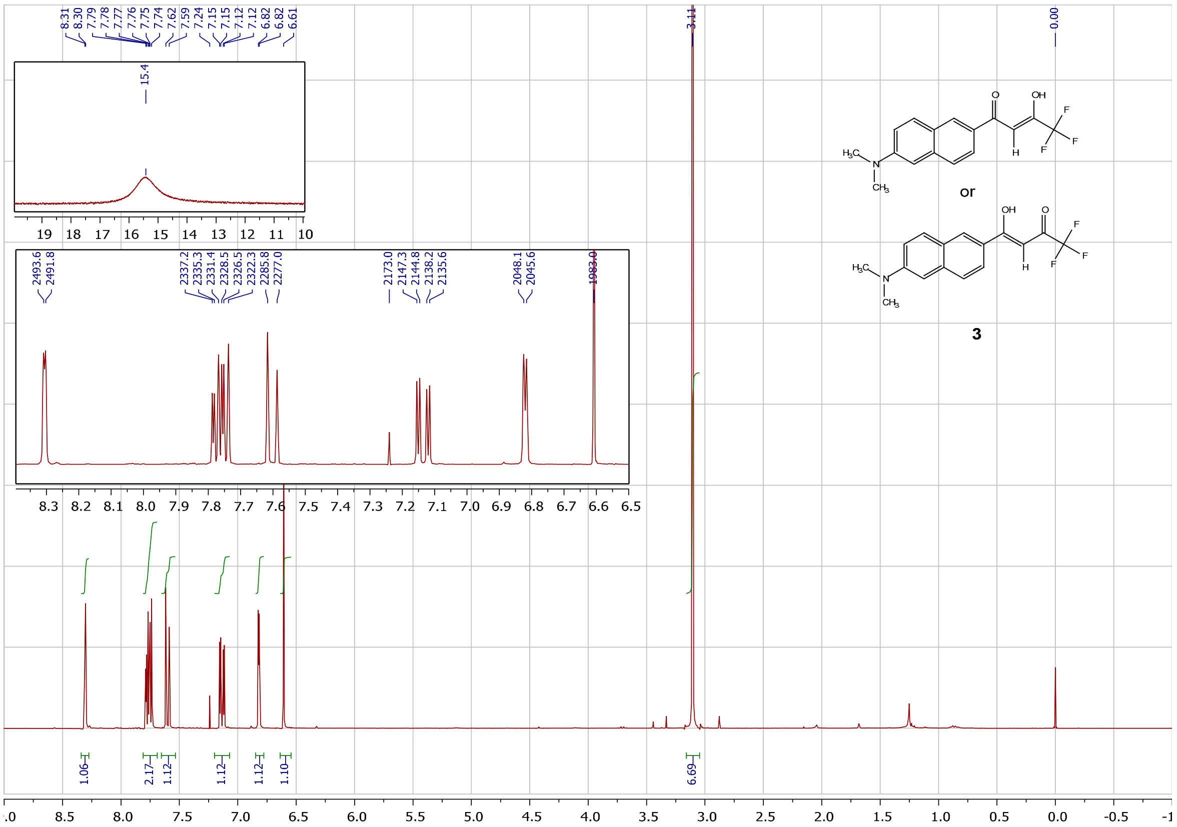

Figure S3. ^{1}H-NMR spectrum of the compound **3**.

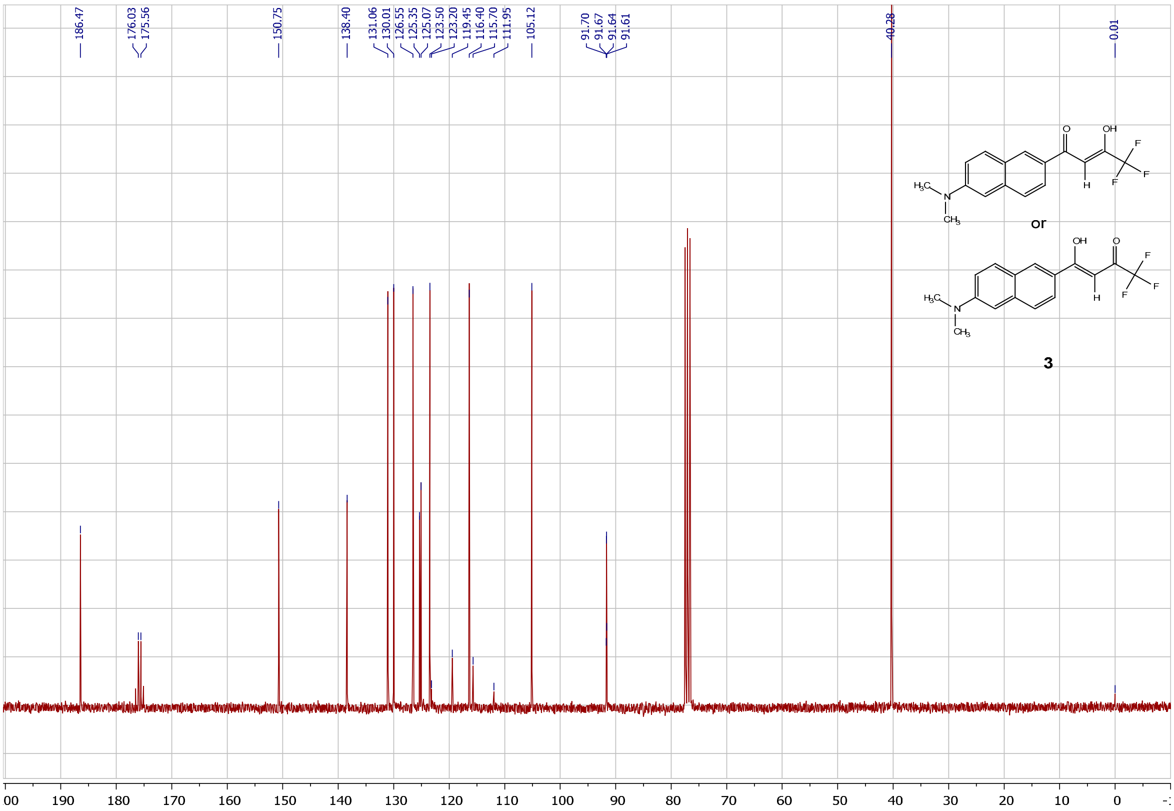

Figure S4. ^{13}C-NMR spectrum of the compound **3**.

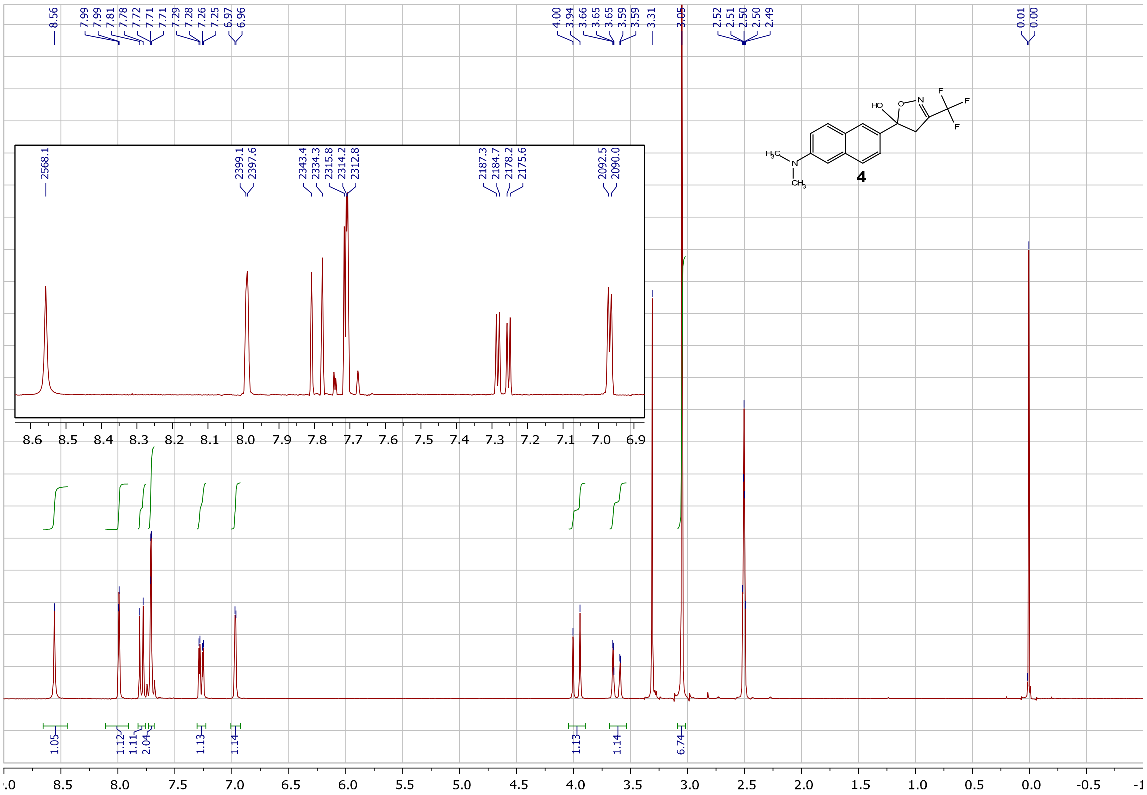

Figure S5. ^{1}H-NMR spectrum of the compound **4**.

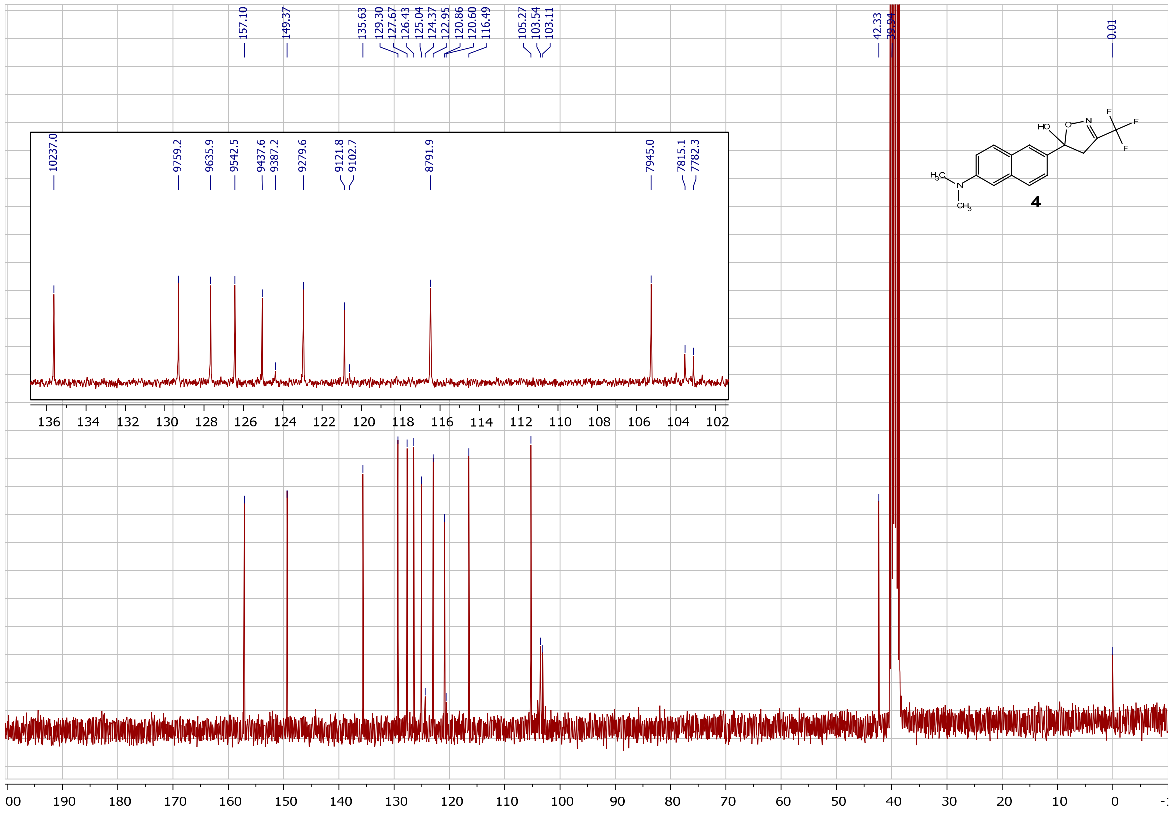

Figure S6. ^{13}C-NMR spectrum of the compound **4**.

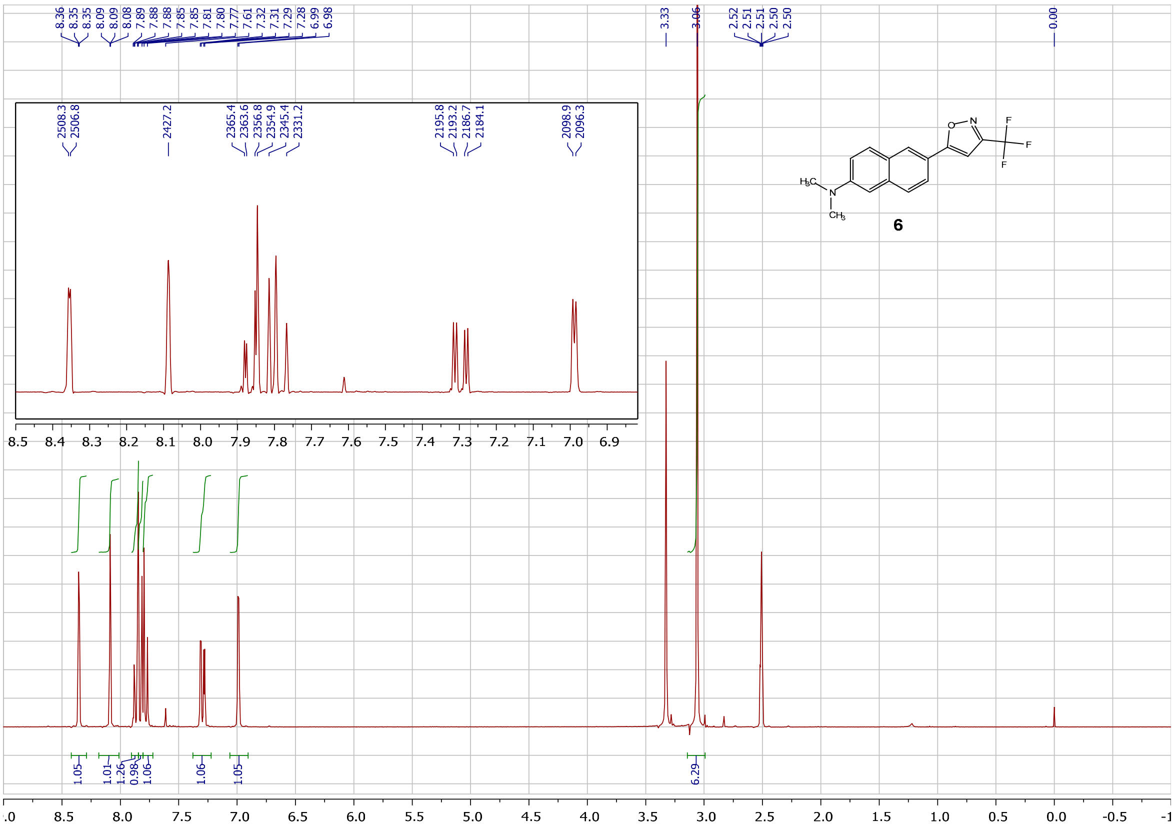

Figure S7. ^{1}H-NMR spectrum of the compound **6**.

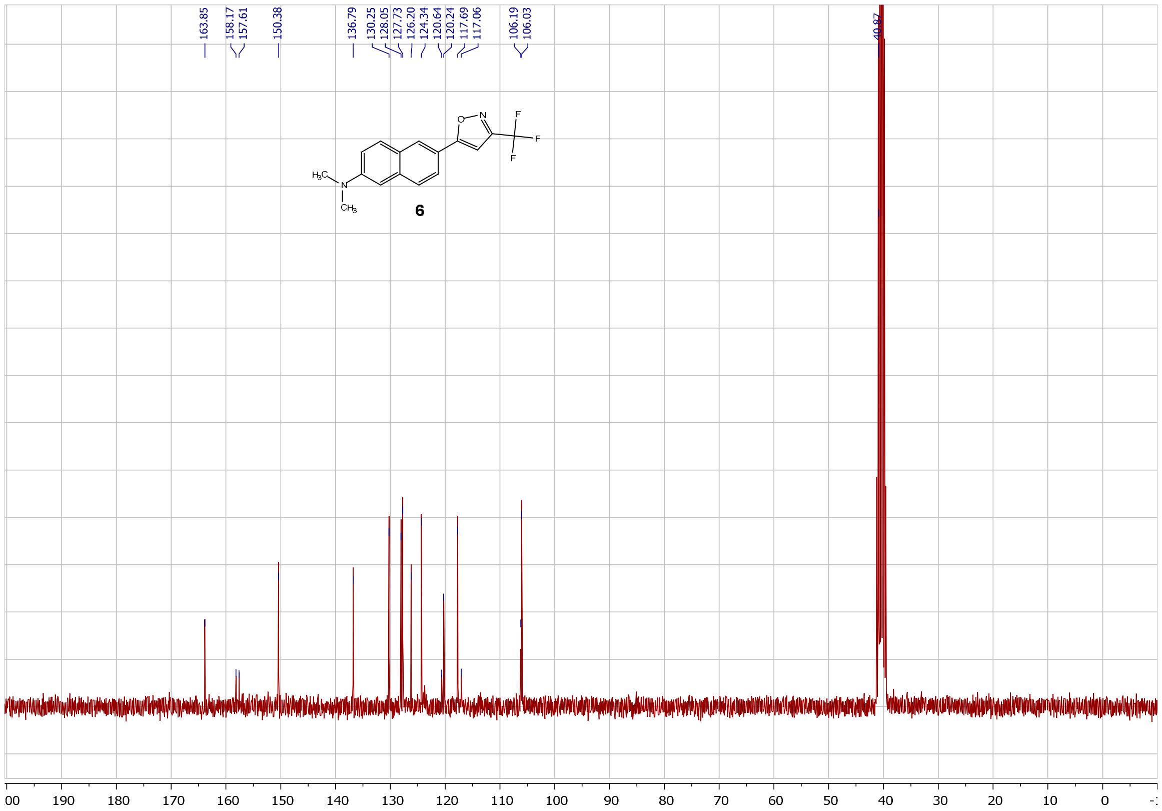

Figure S8. ^{13}C-NMR spectrum of the compound **6**.

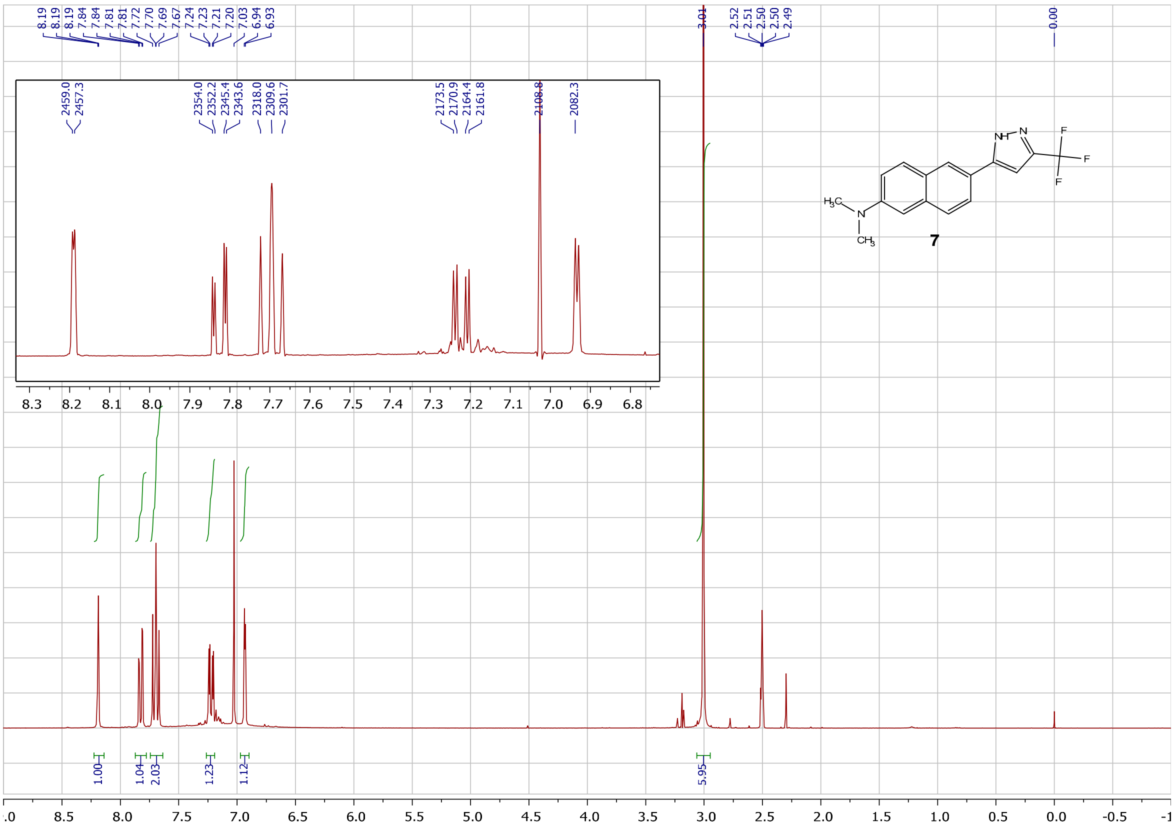

Figure S9. ^{1}H-NMR spectrum of the compound **7**.

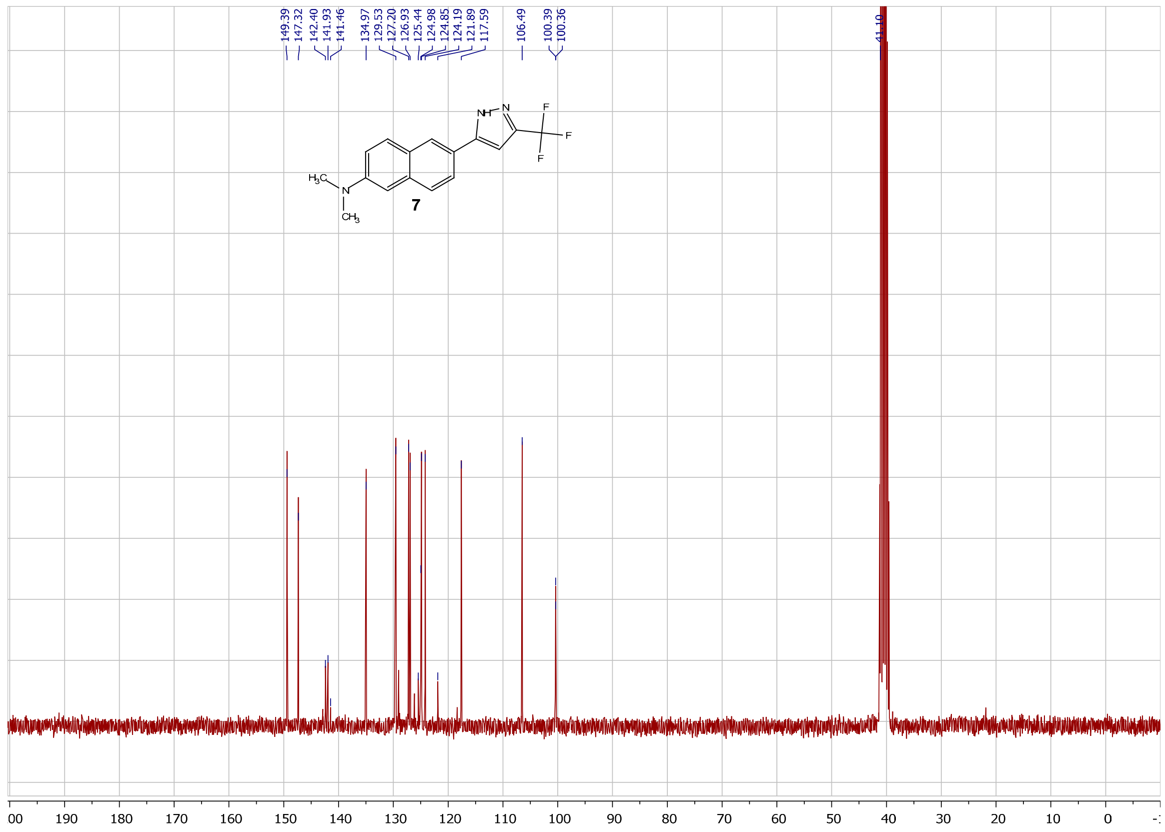

Figure S10. ^{13}C-NMR spectrum of the compound **7**.

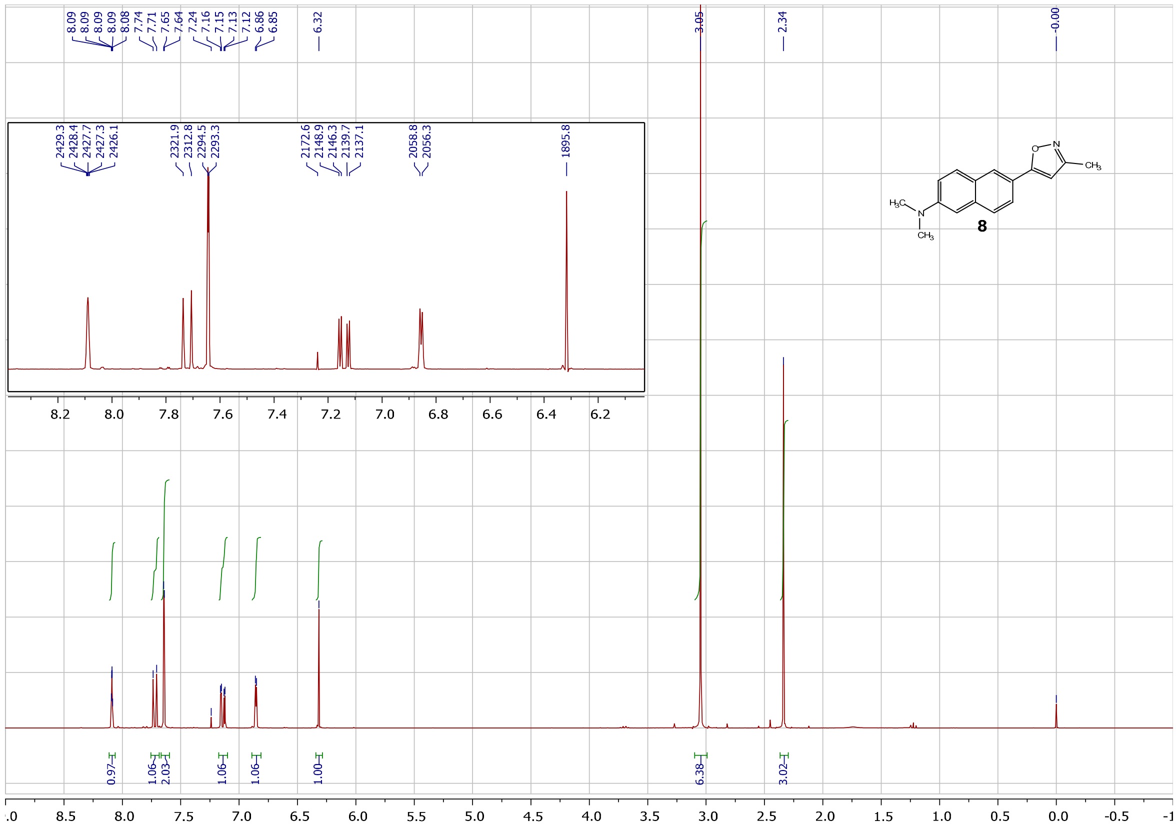

Figure S11. ^{1}H-NMR spectrum of the compound **8**.

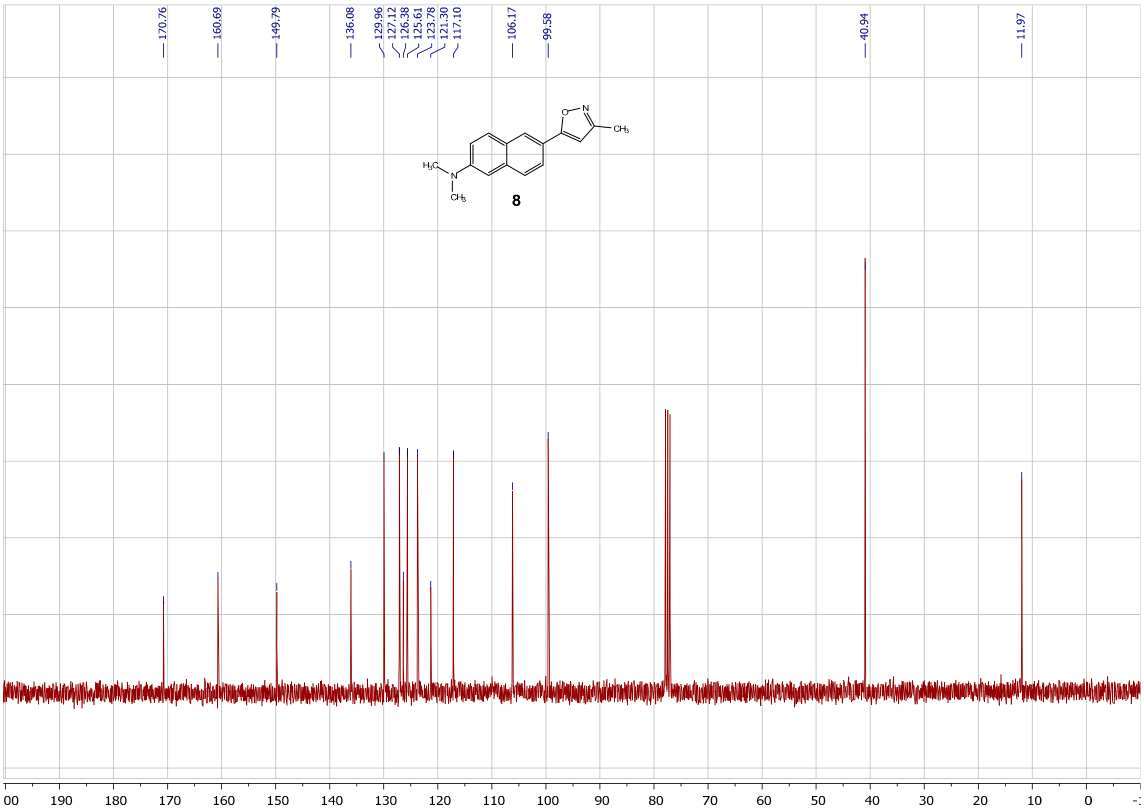

Figure S12. ^{13}C-NMR spectrum of the compound **8**.

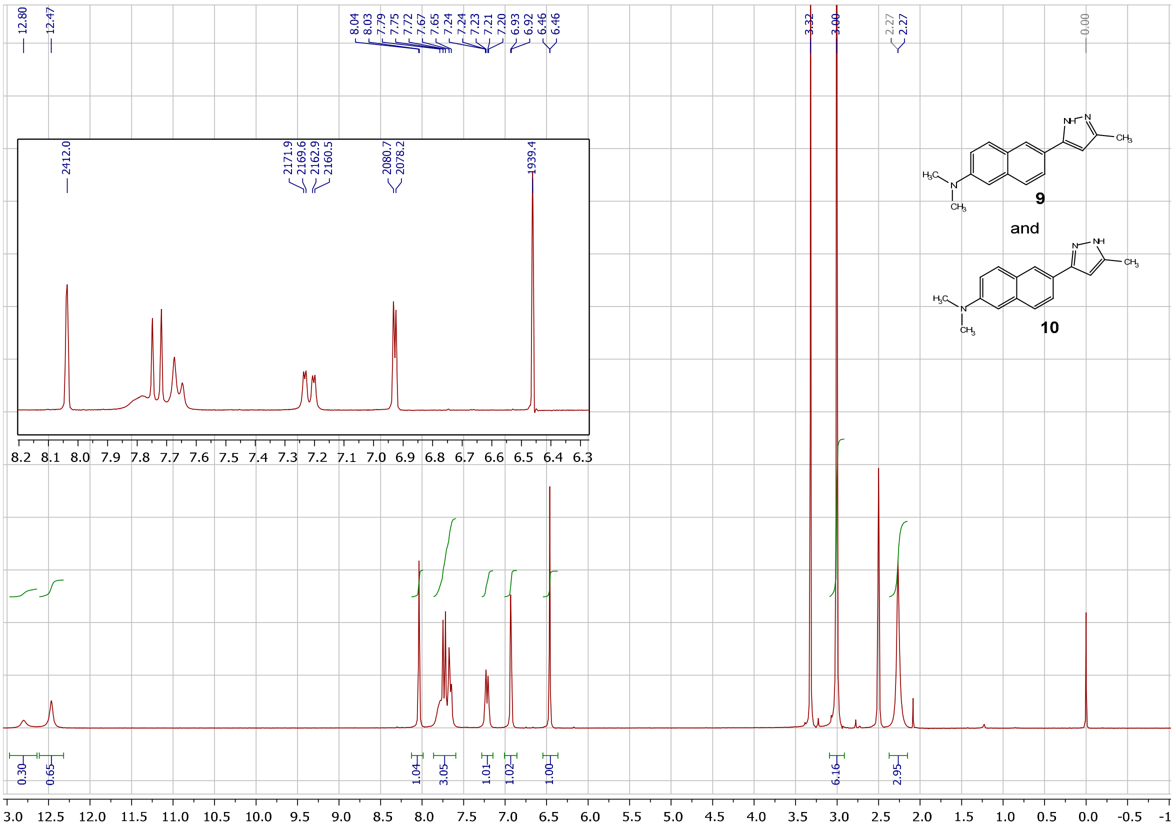

Figure S13. ^{1}H-NMR spectrum of the compounds **9** and **10**.

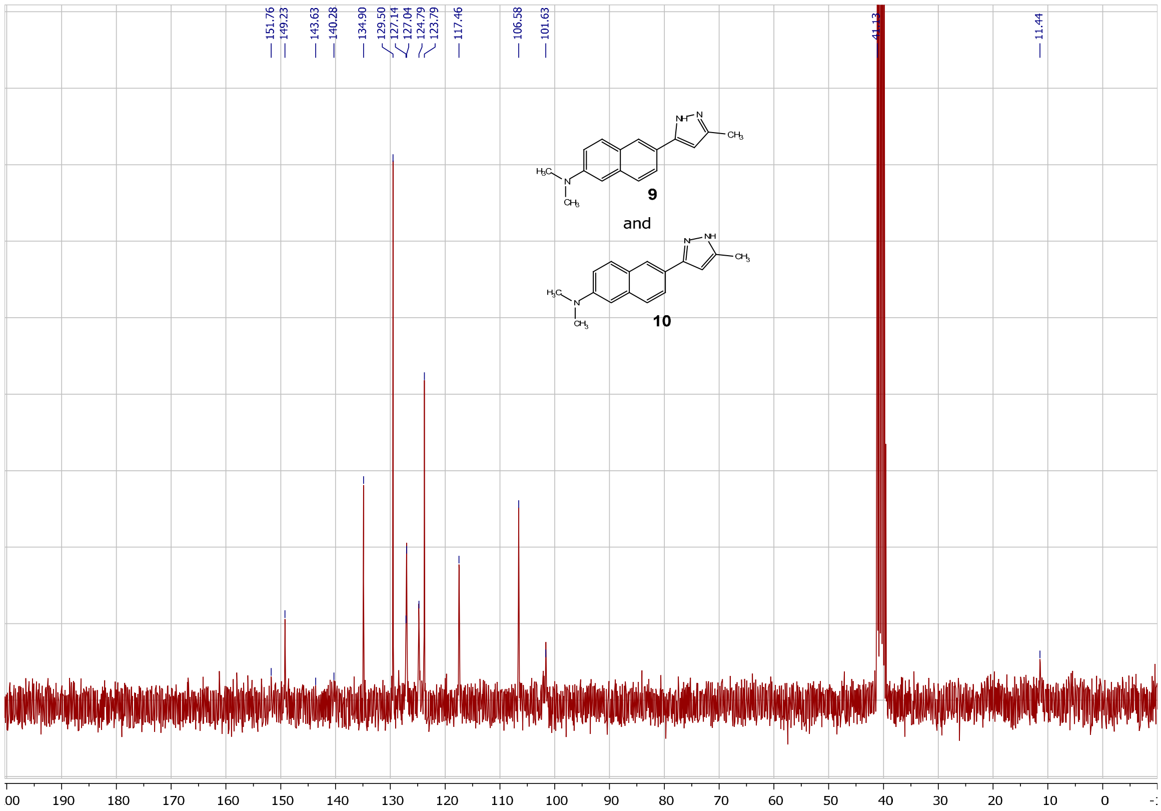

Figure S14. ^{13}C-NMR spectrum of the compounds **9** and **10**.

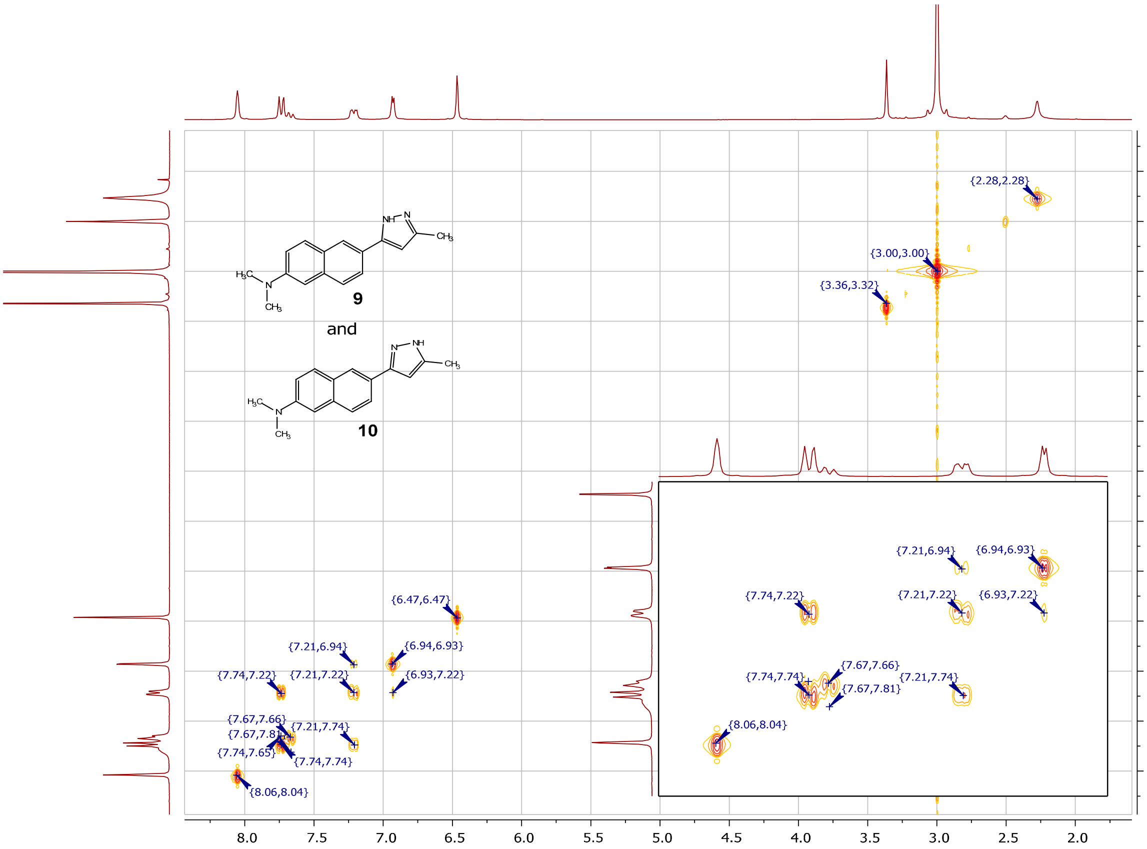

Figure S15. gs-COSY NMR spectrum of the compounds **9** and **10.**

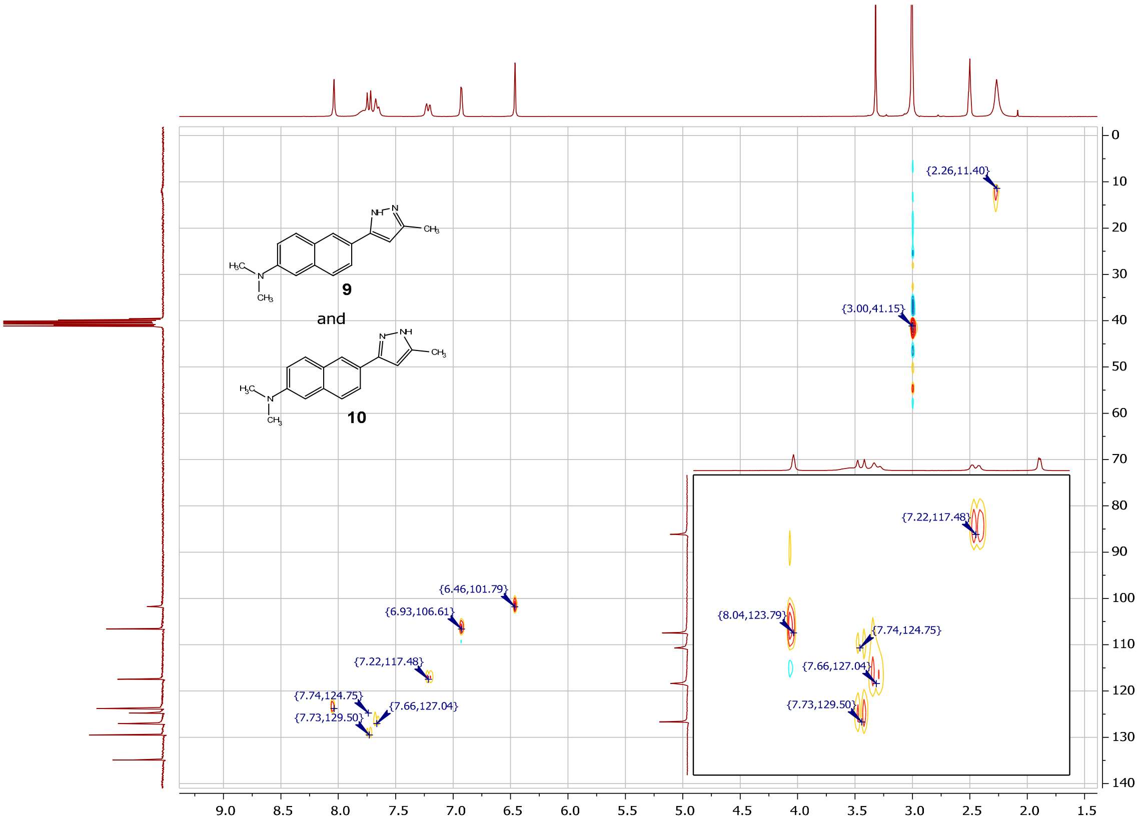

Figure S16. gs-HSQC NMR spectrum of the compounds **9** and **10.**

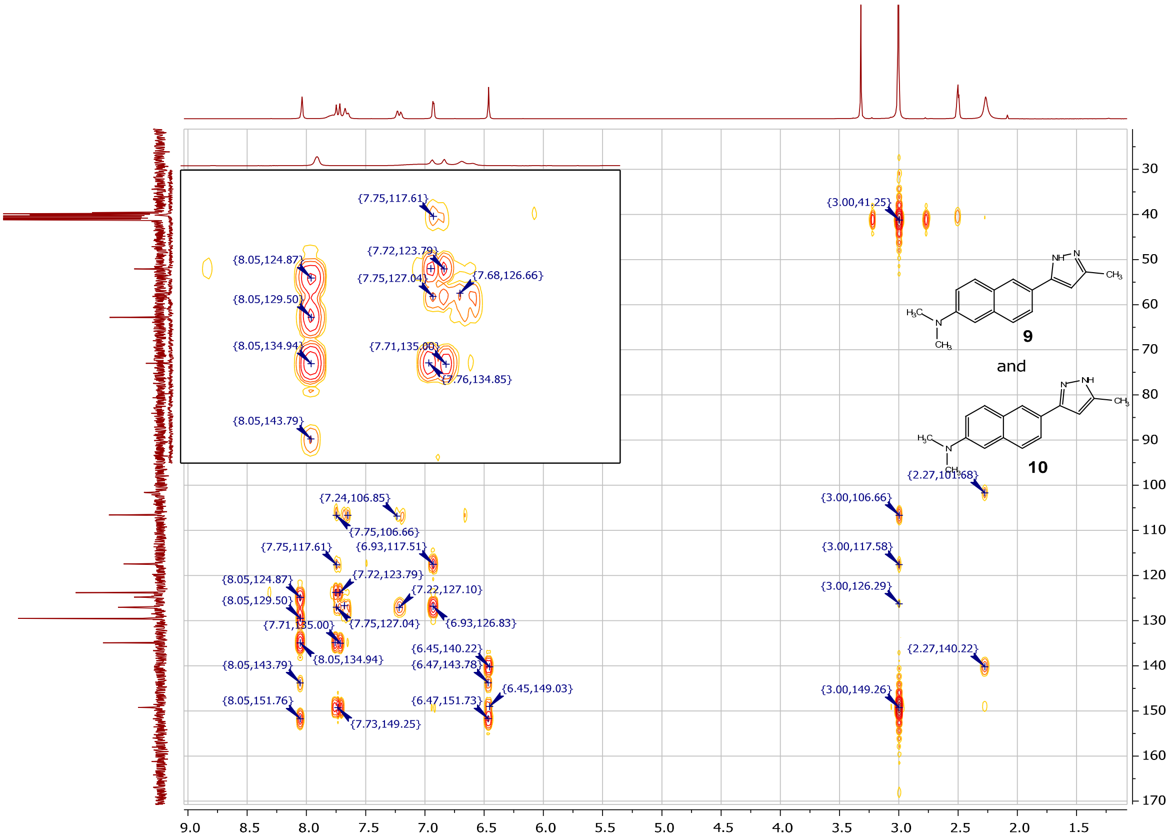

Figure S17. gs-HMBC NMR spectrum of the compounds **9** and **10.**

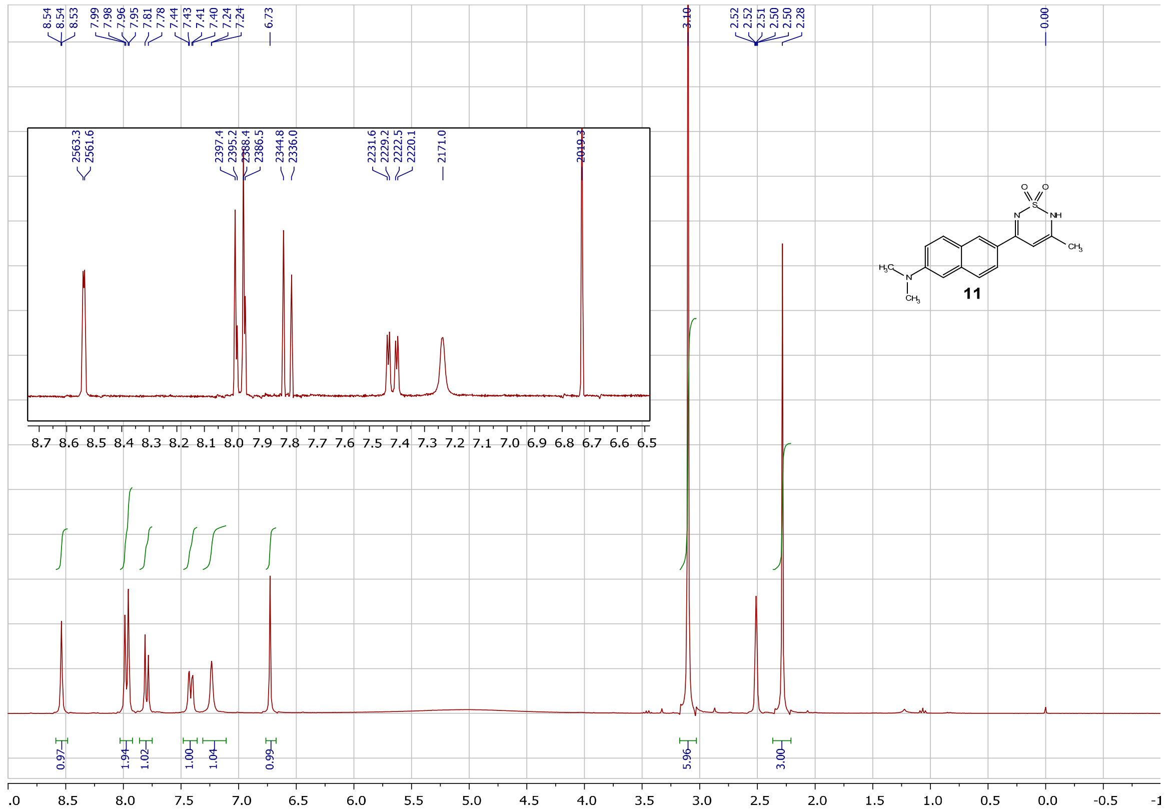

Figure S18. ^{1}H-NMR spectrum of the compound **11**.

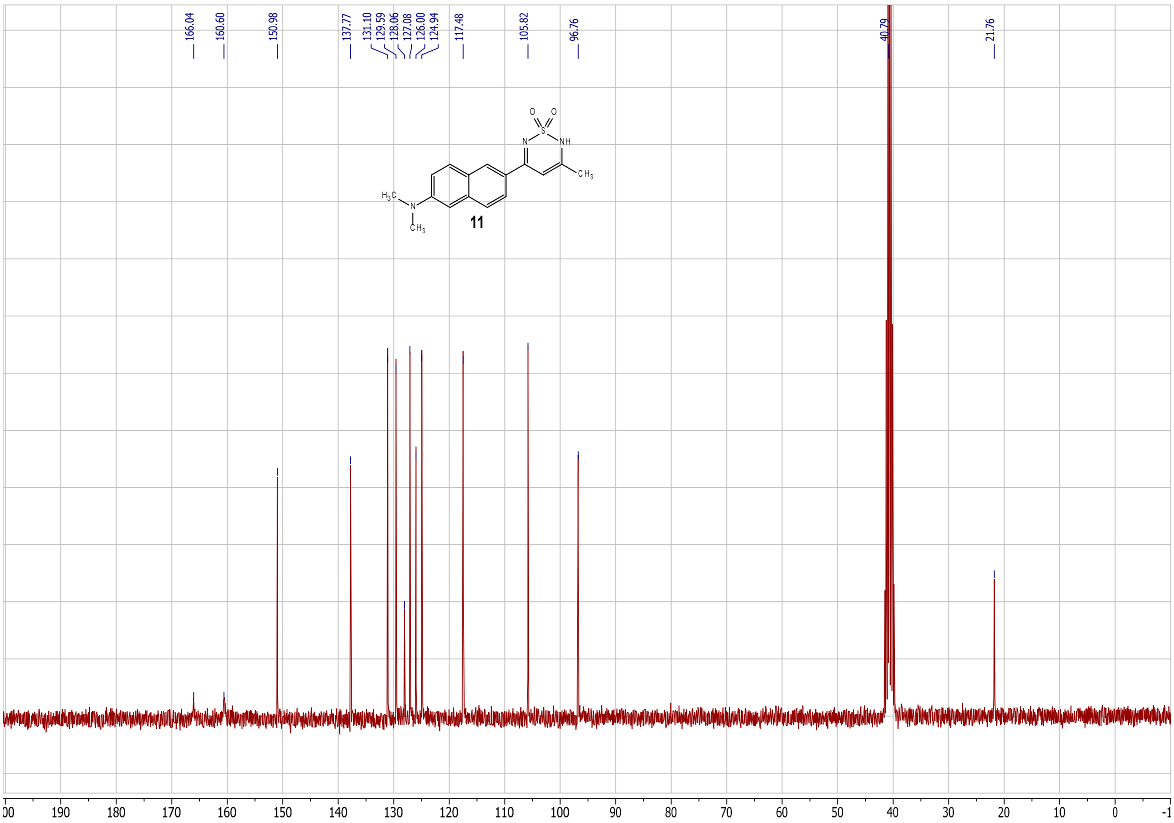

Figure S19. ^{13}C-NMR spectrum of the compound **11**.

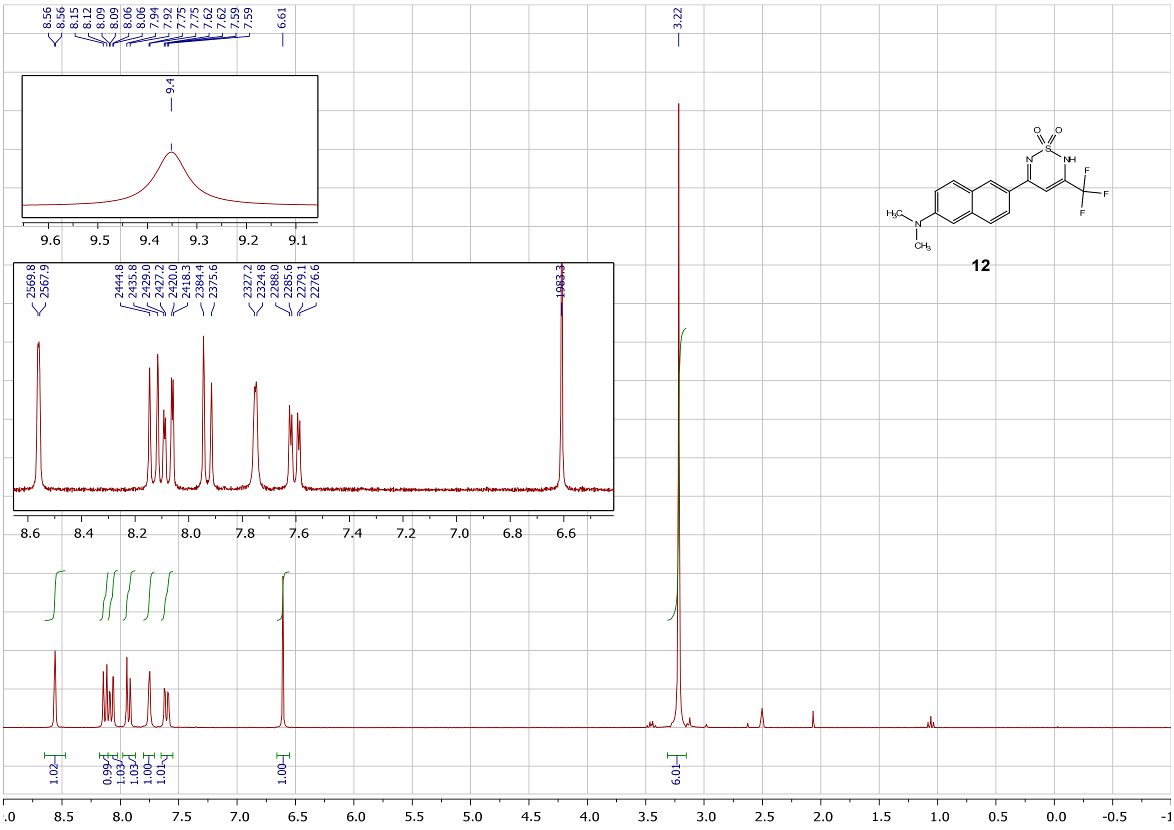

Figure S20. ^{1}H-NMR spectrum of the compound **12**.

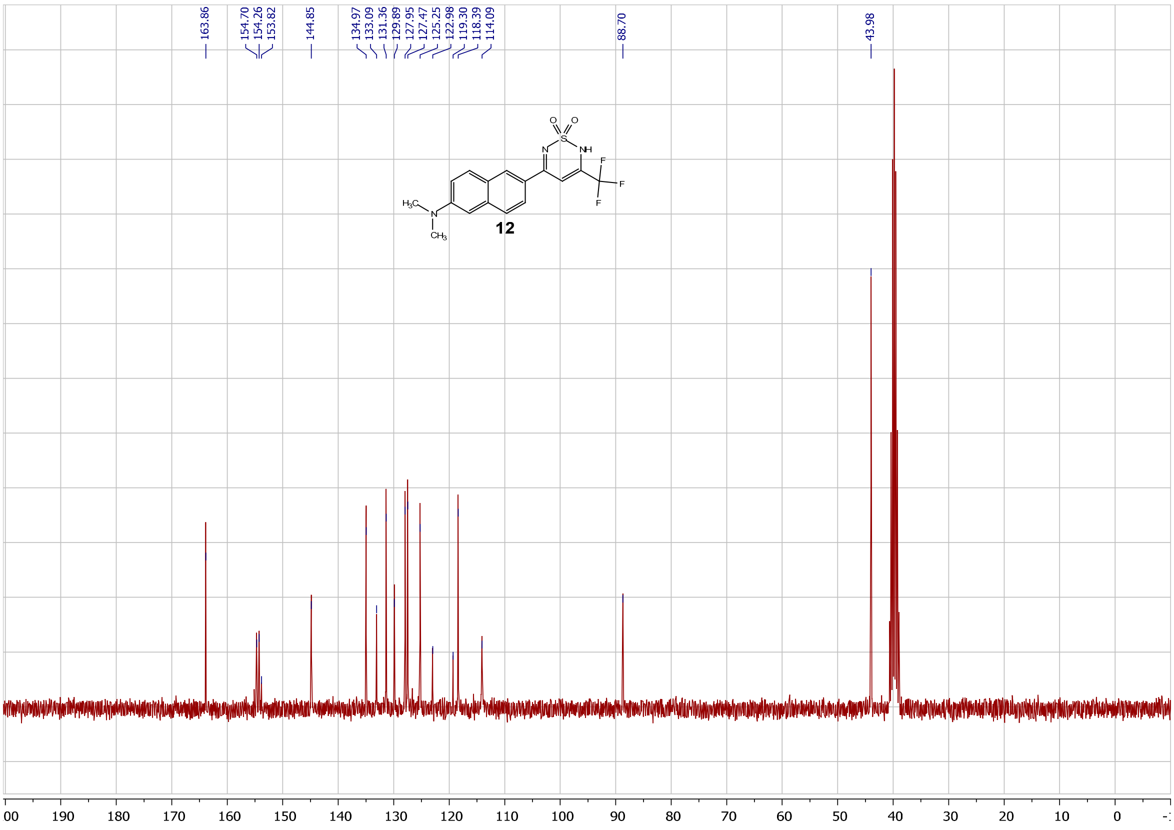

Figure S21. ^{13}C-NMR spectrum of the compound **12**.

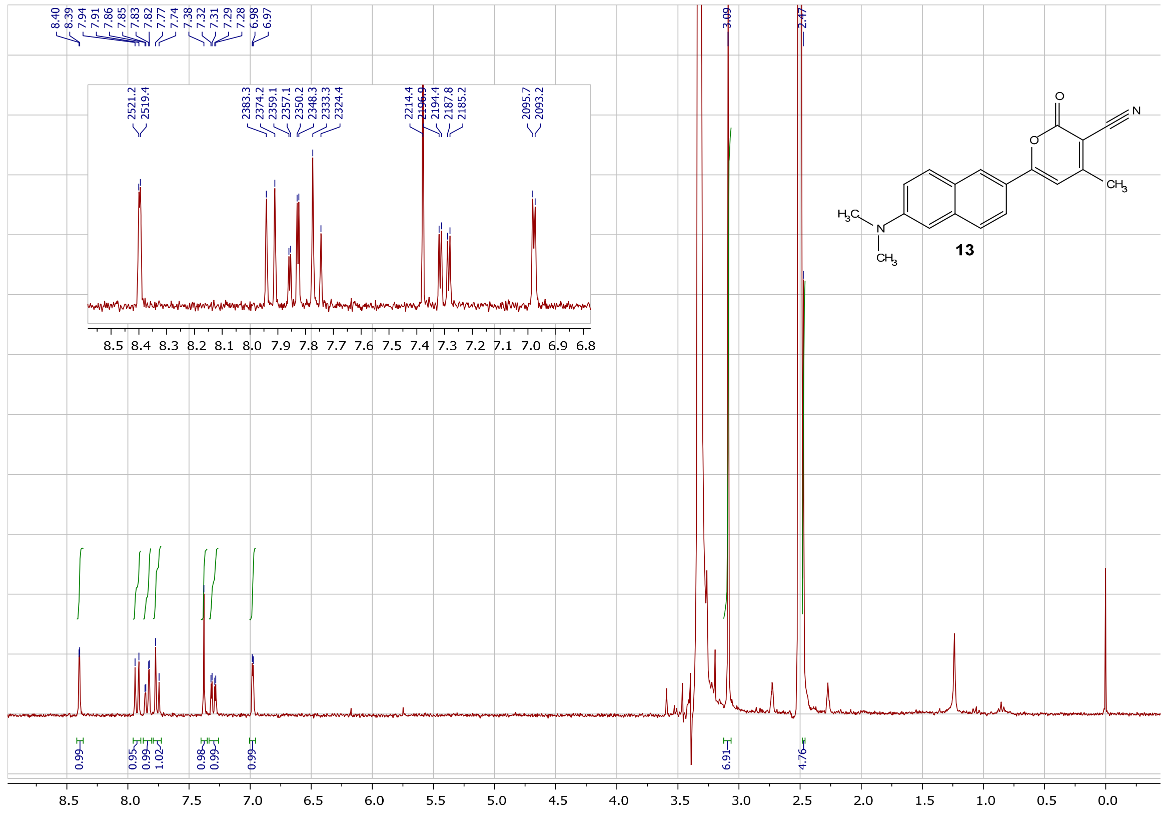

Figure S22. ^{1}H-NMR spectrum of the compound **13**.

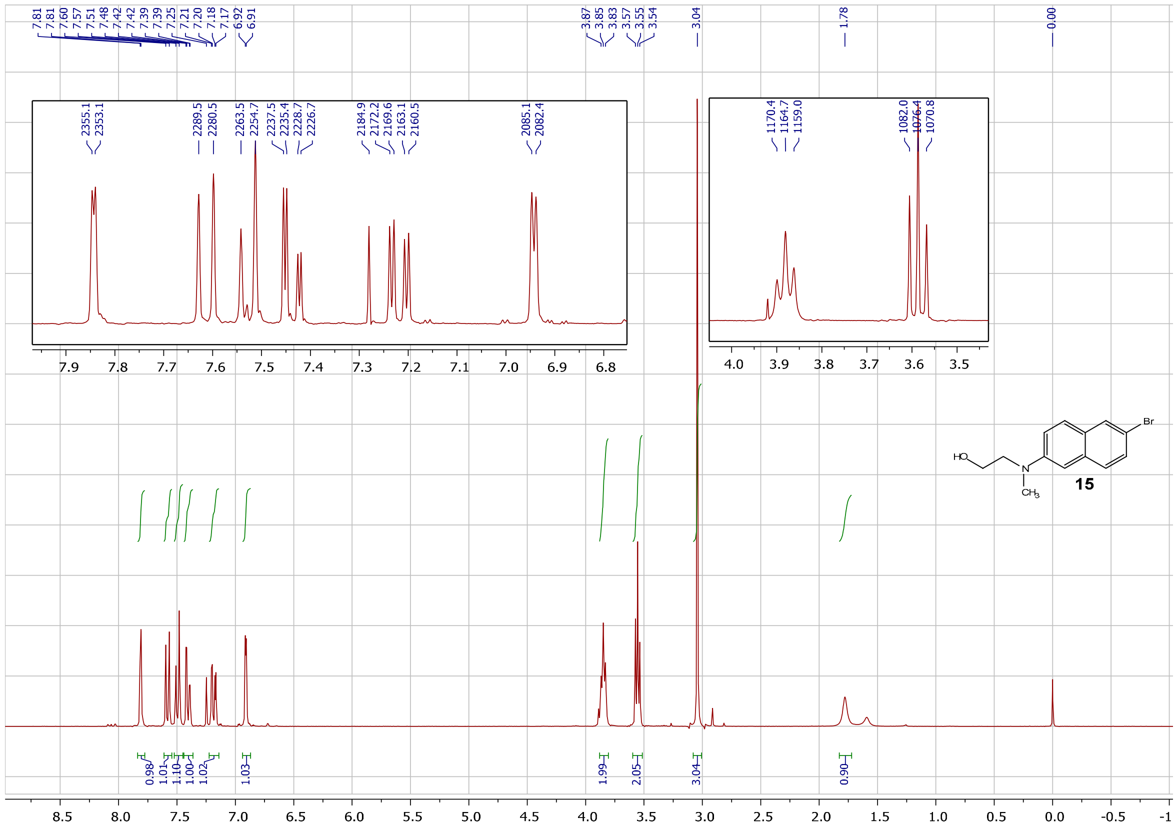

Figure S23. ^{1}H-NMR spectrum of the compound **15**.

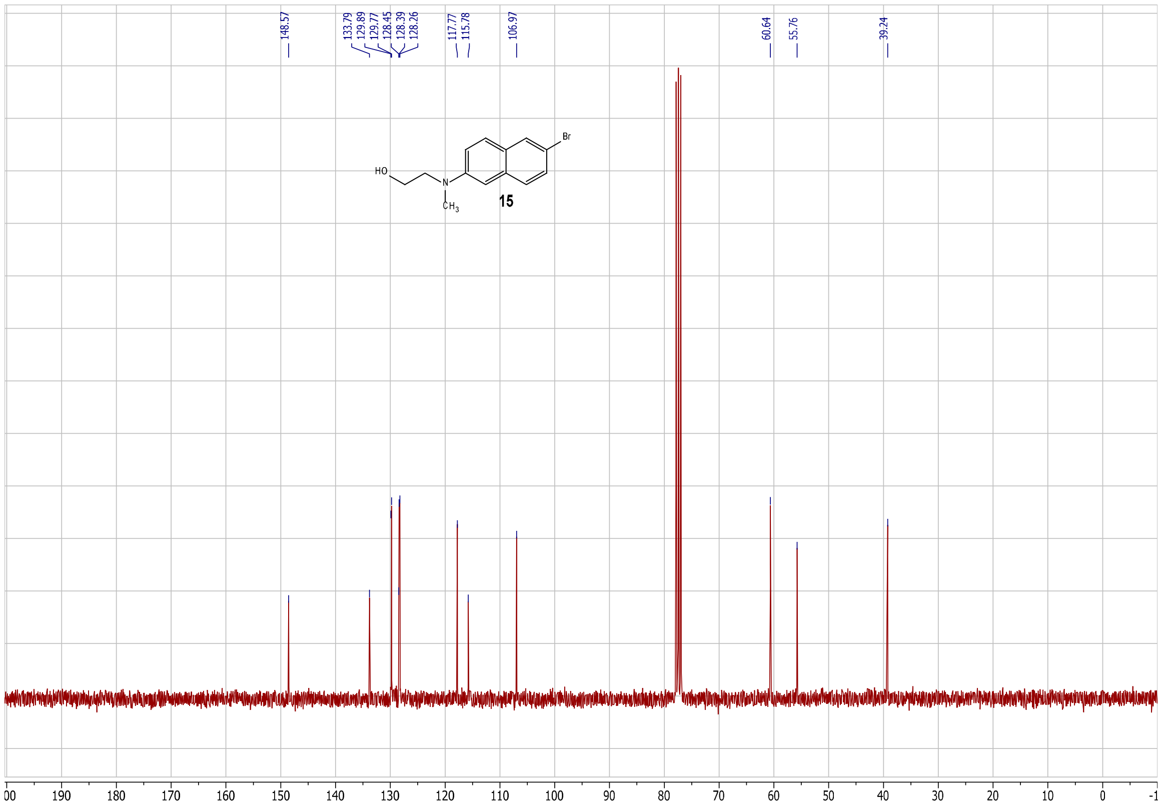

Figure S24. ^{13}C-NMR spectrum of the compound **15**.

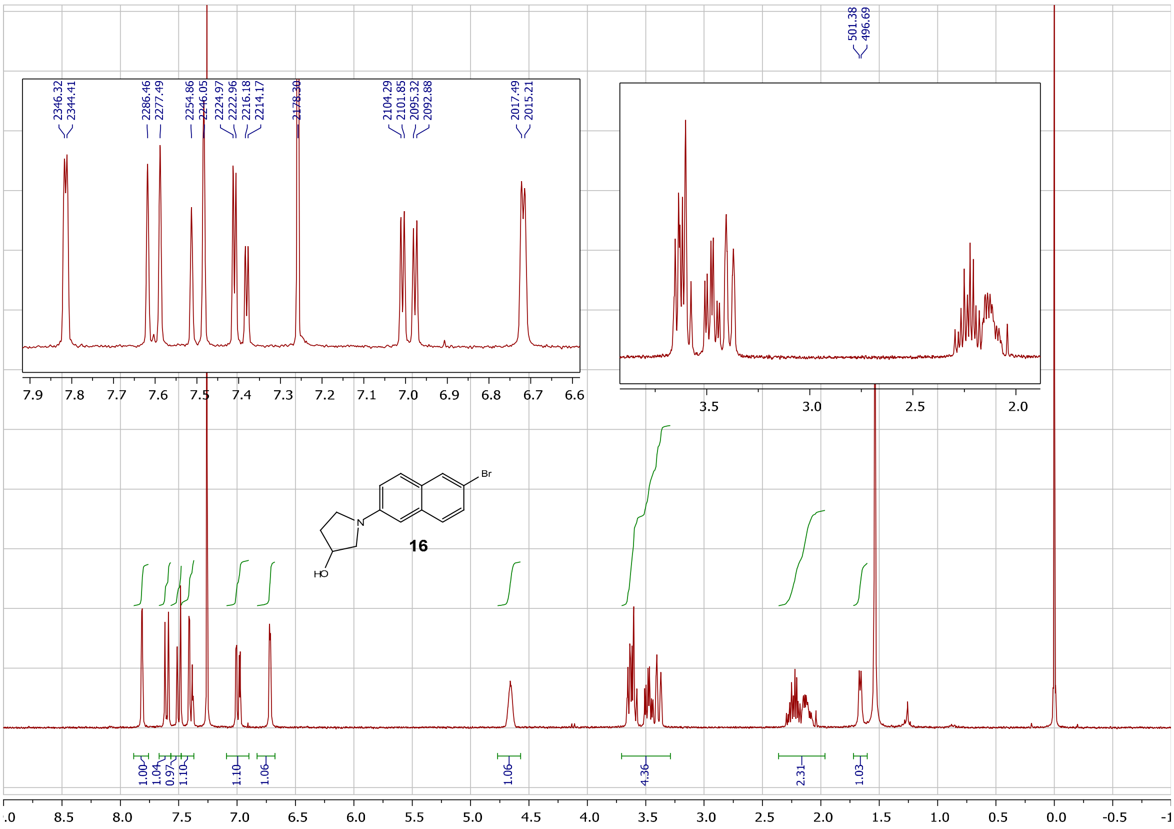

Figure S25. ^{1}H-NMR spectrum of the compound **16**.

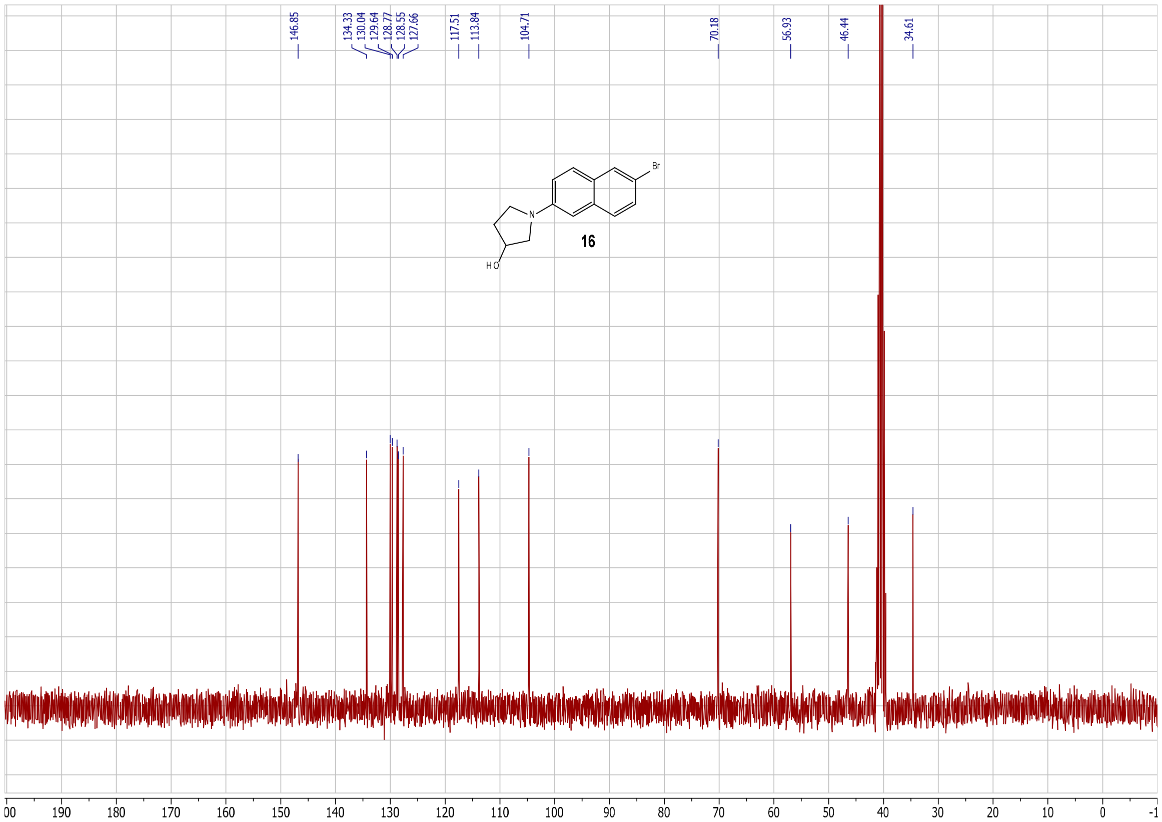

Figure S26. ^{13}C-NMR spectrum of the compound **16**.

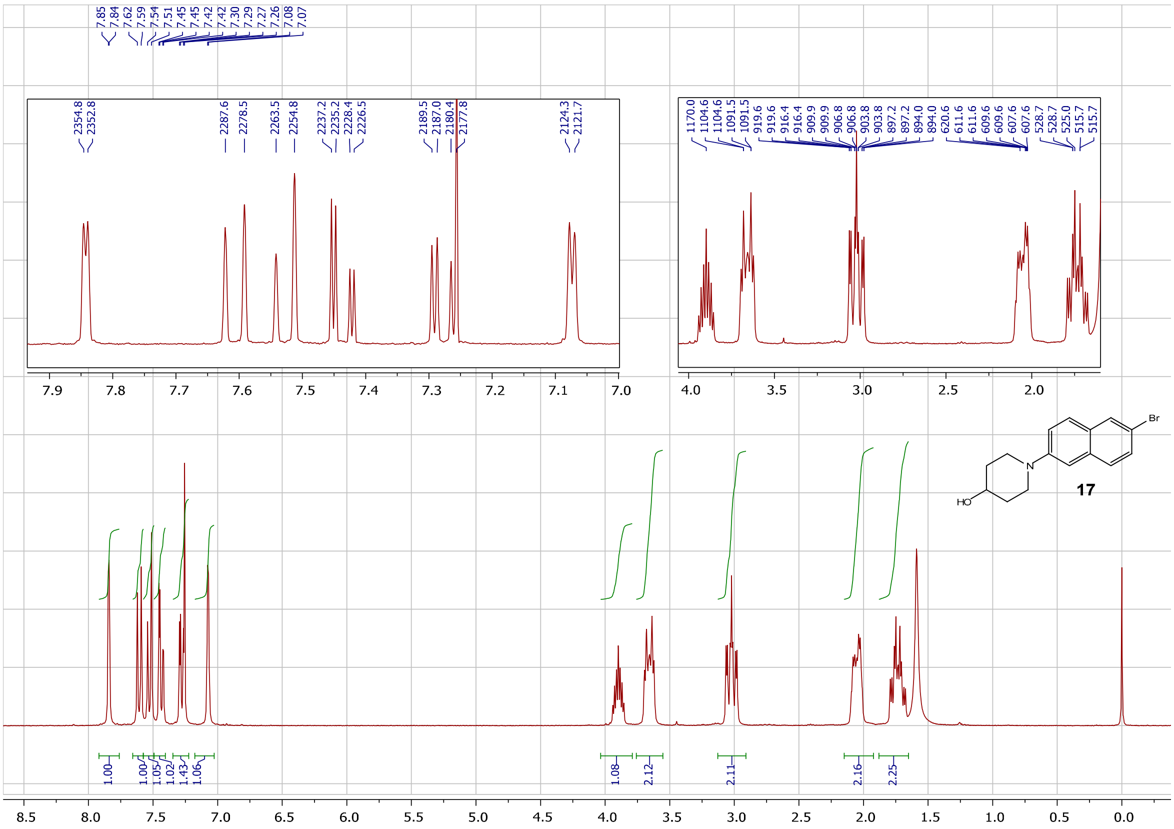

Figure S27. ^{1}H-NMR spectrum of the compound **17**.

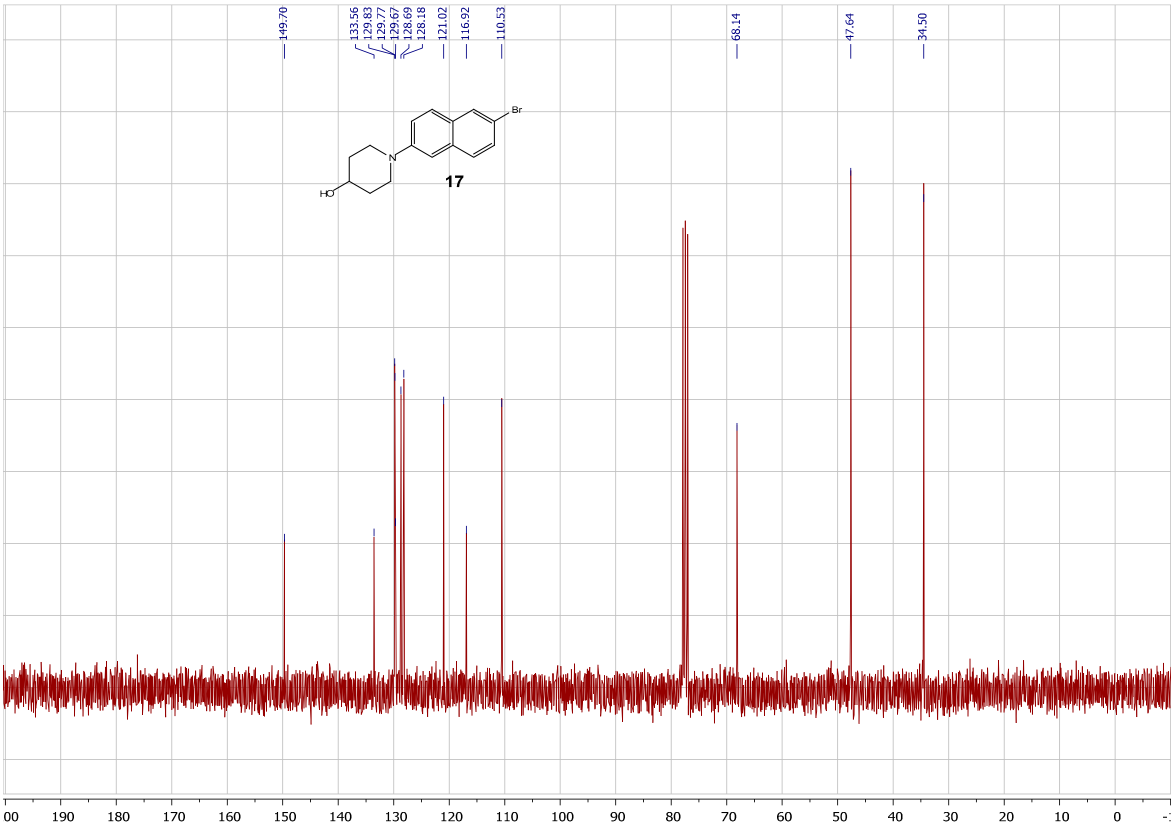

Figure S28. ^{13}C-NMR spectrum of the compound **17**.

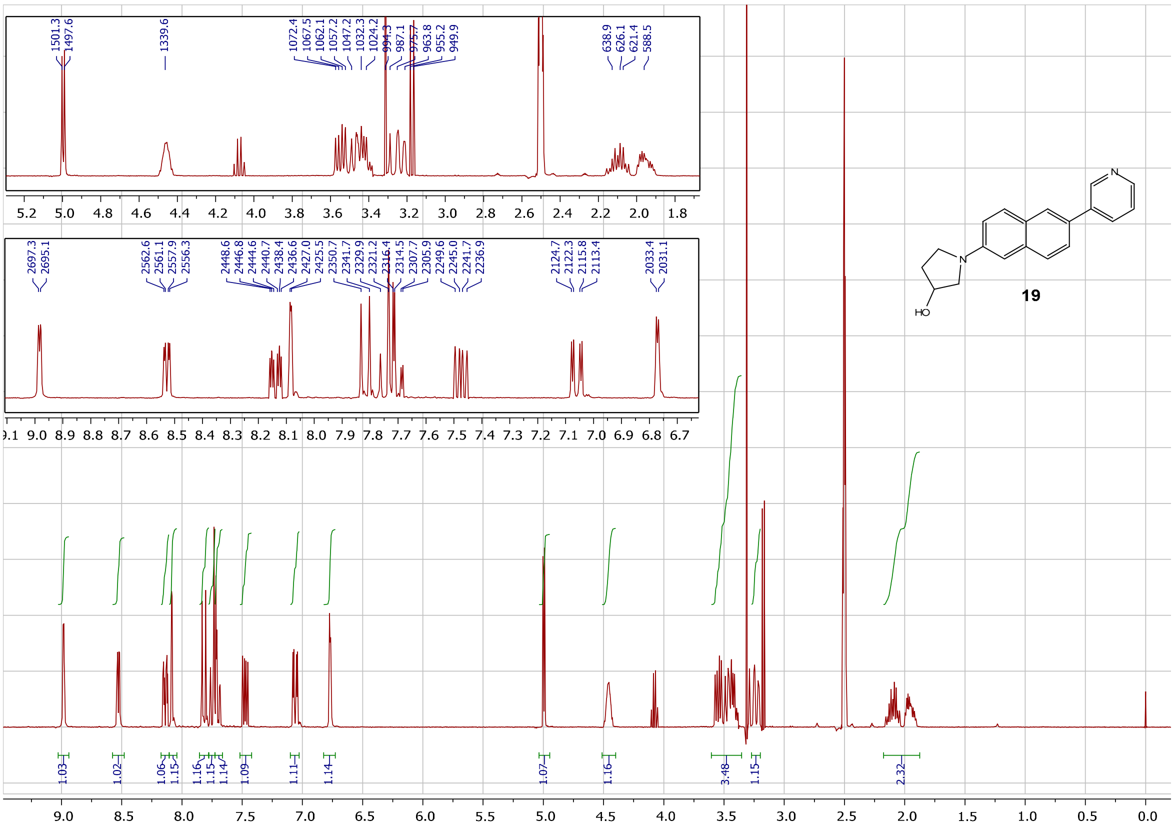

Figure S29. ^{1}H-NMR spectrum of the compound **19**.

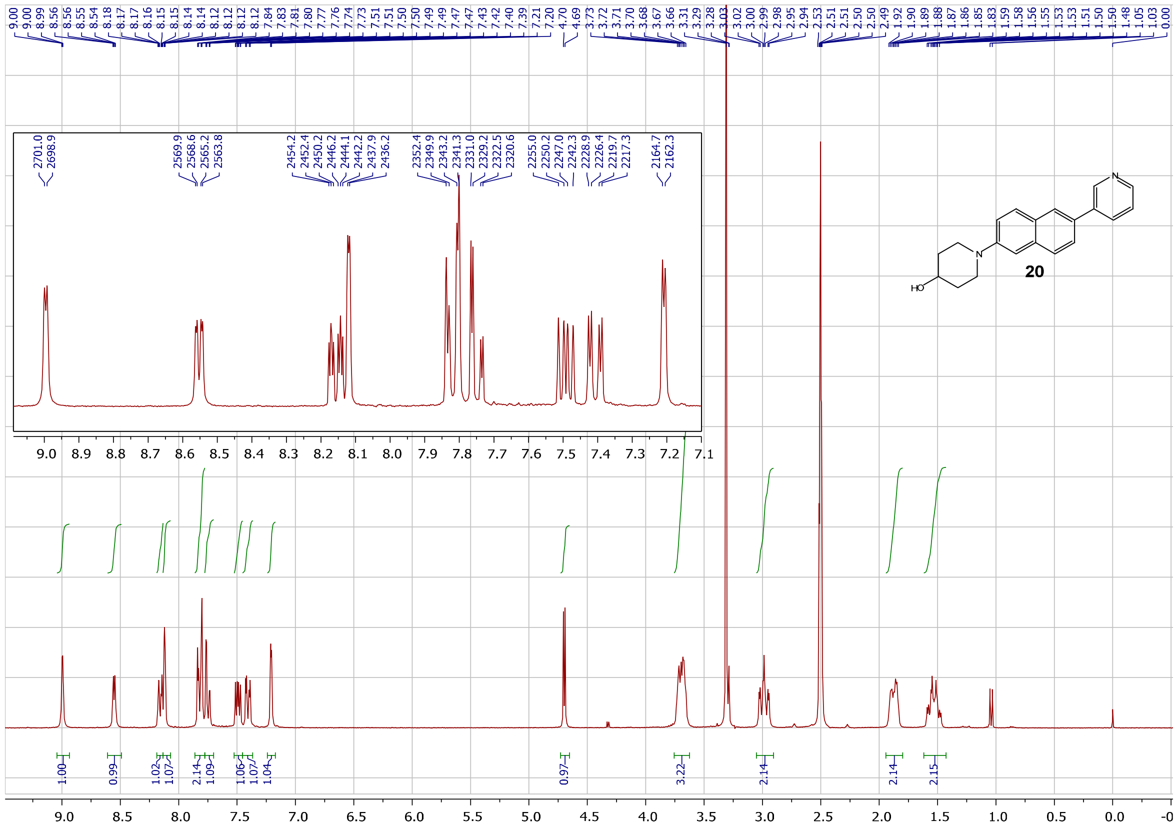

Figure S30. ^{1}H-NMR spectrum of the compound **20**.

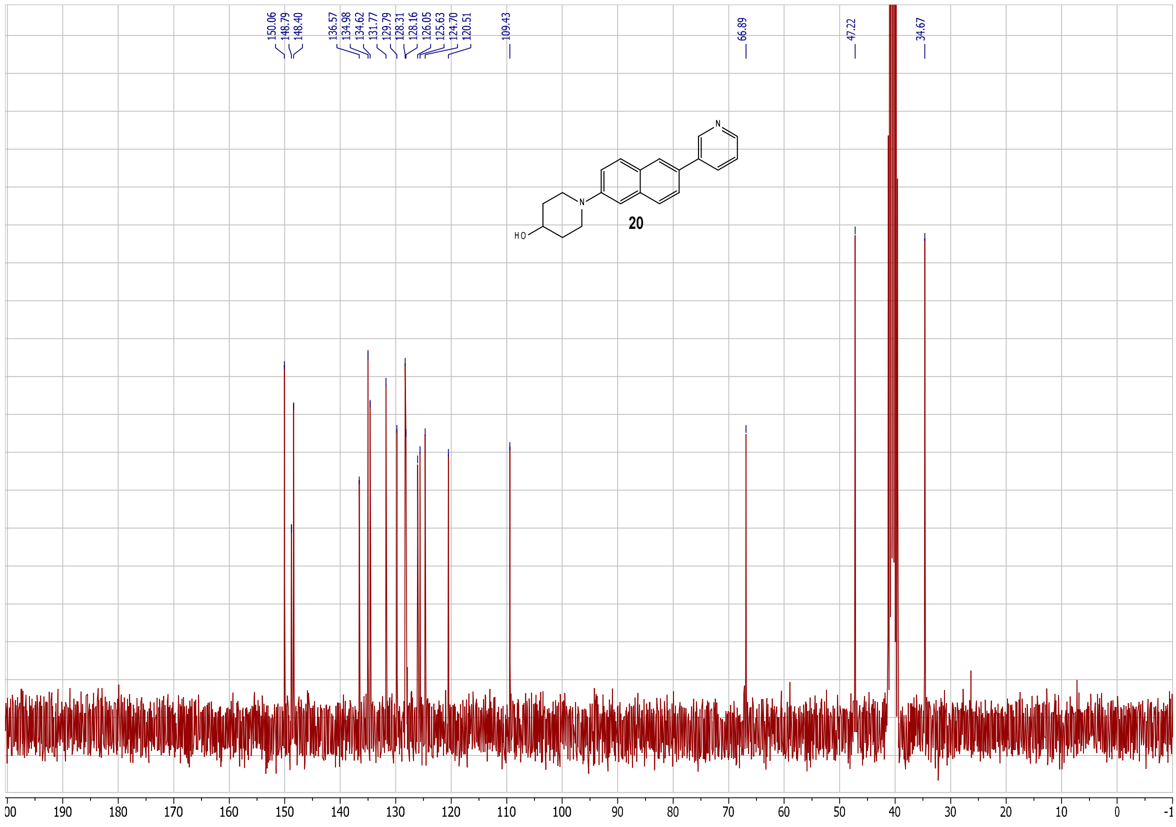

Figure S31. ^{13}C-NMR spectrum of the compound **20**.

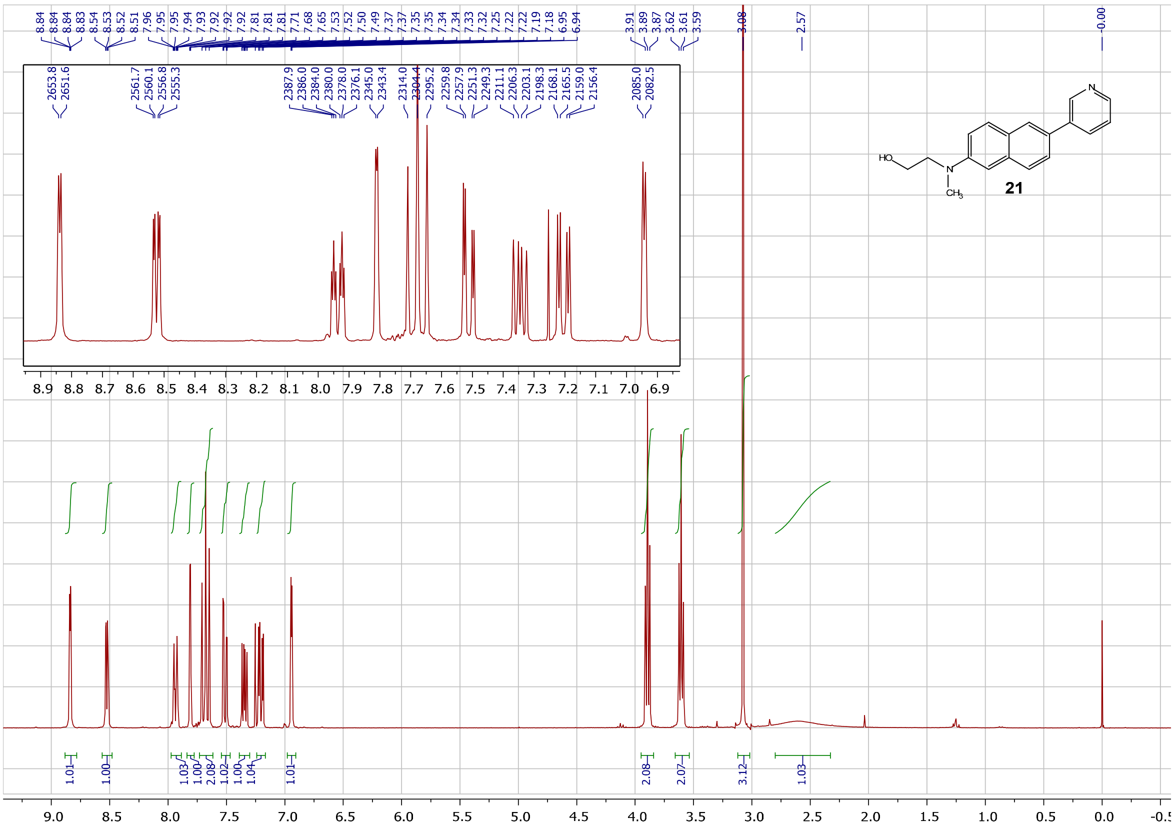

Figure S32. ^{1}H-NMR spectrum of the compound **21**.

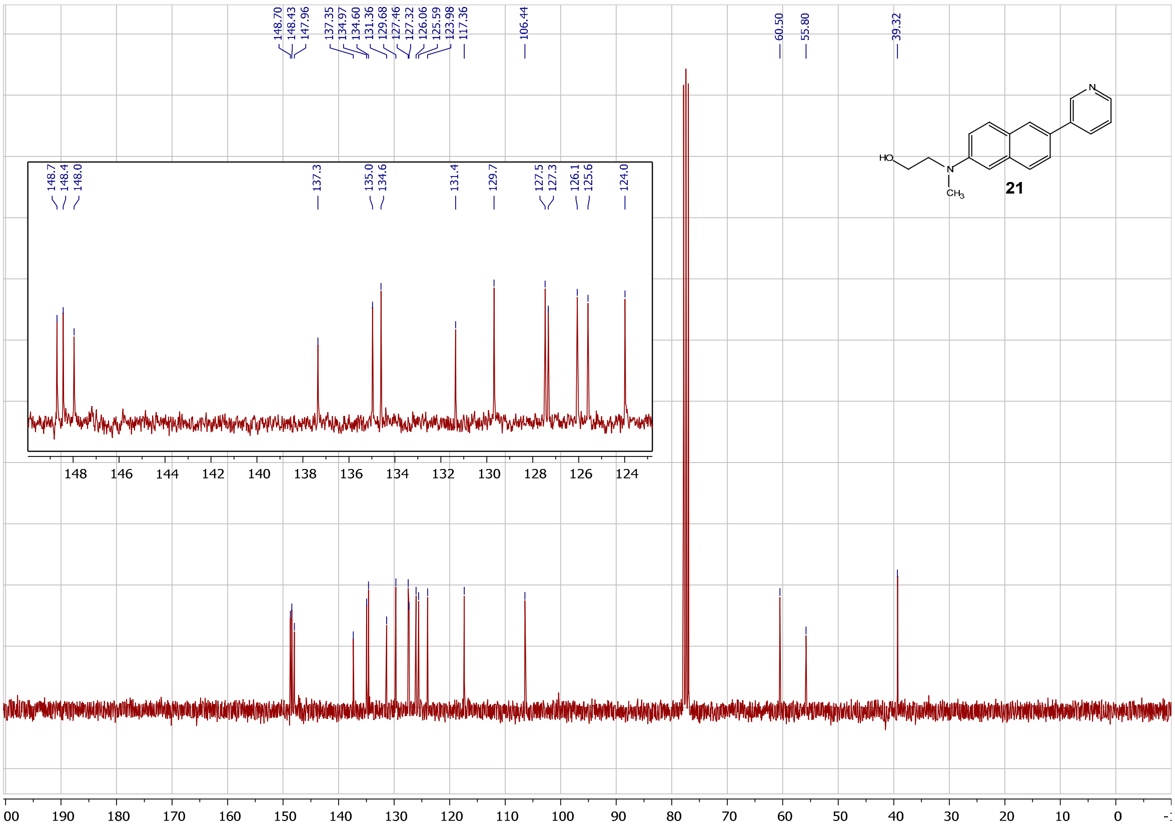

Figure S33. ^{13}C-NMR spectrum of the compound **21**.

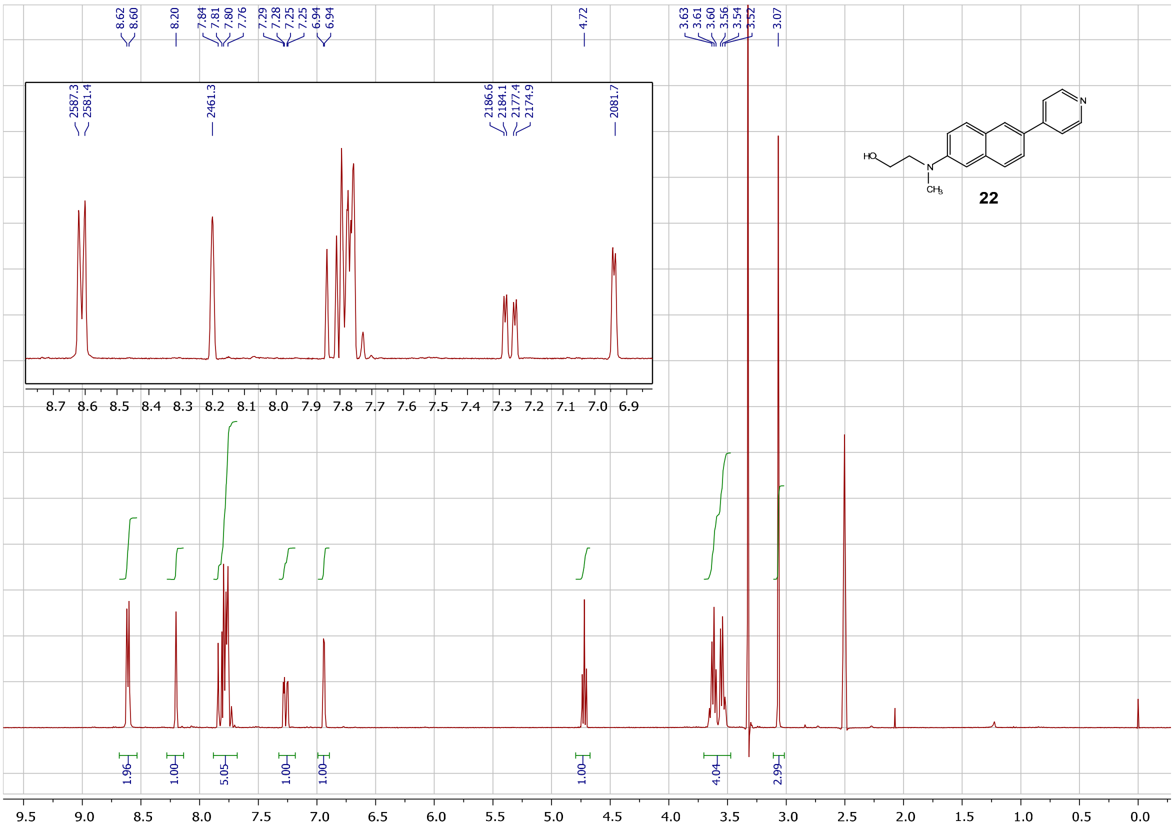

Figure S34. ^{1}H-NMR spectrum of the compound **22**.

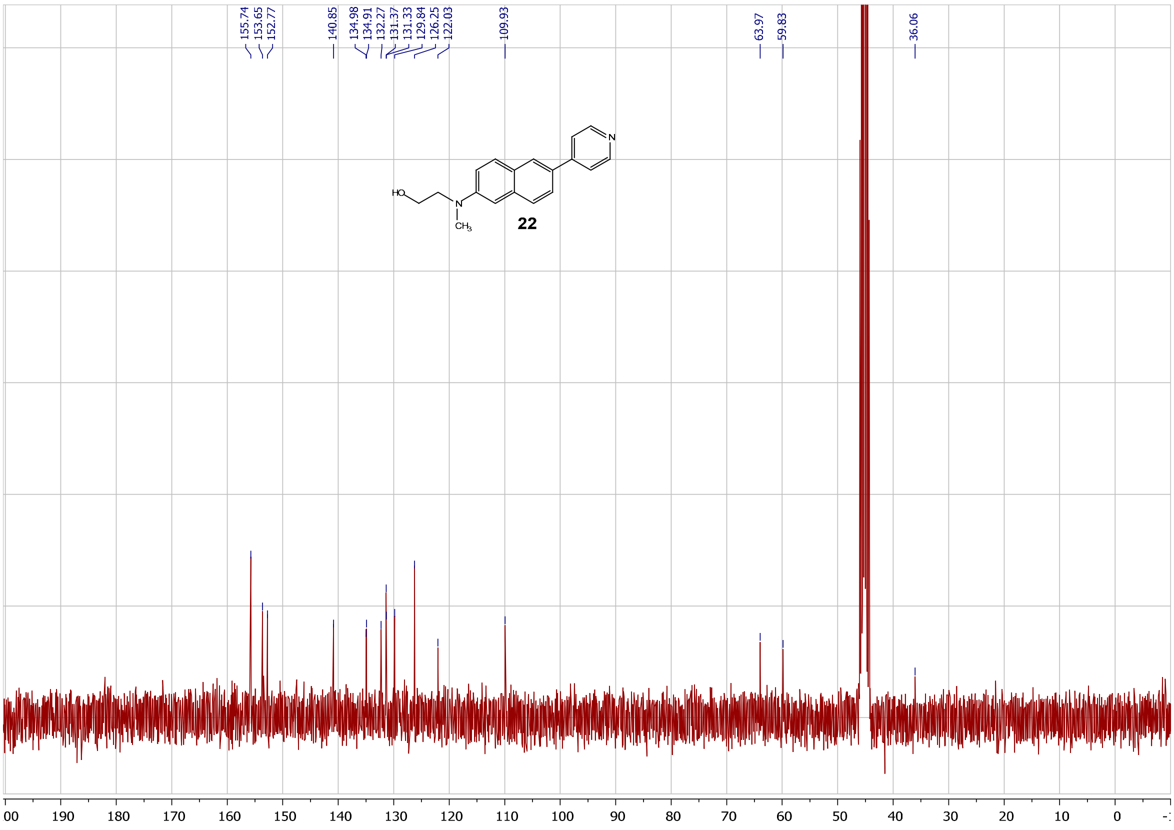

Figure S35. ^{13}C-NMR spectrum of the compound **22**.

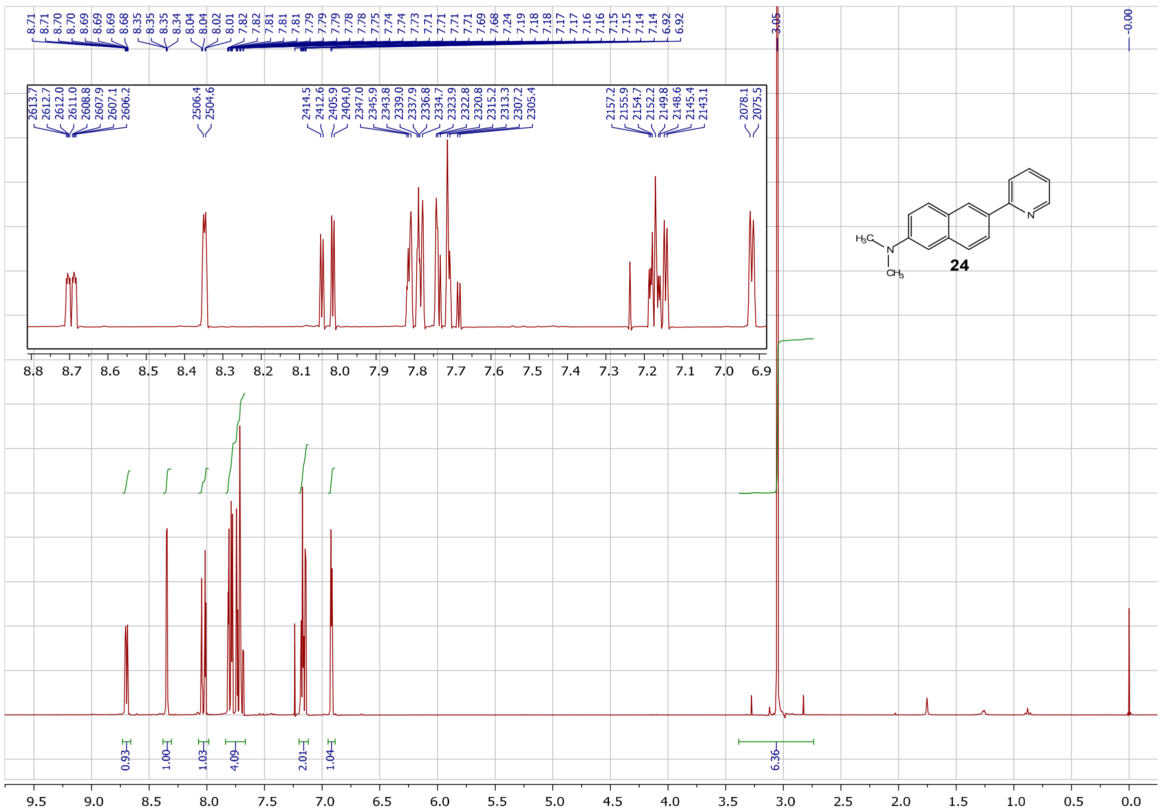

Figure S36. ^{1}H-NMR spectrum of the compound **24**.

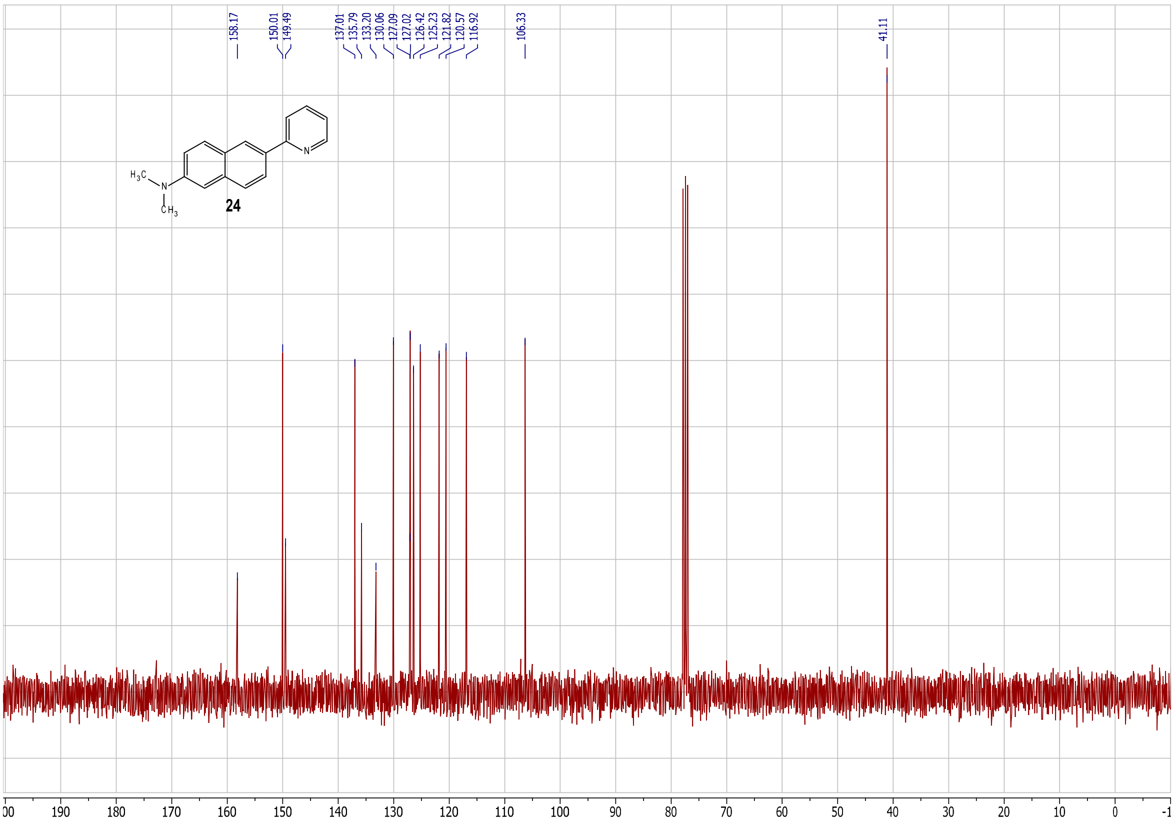

Figure S37. ^{13}C-NMR spectrum of the compound **24**.

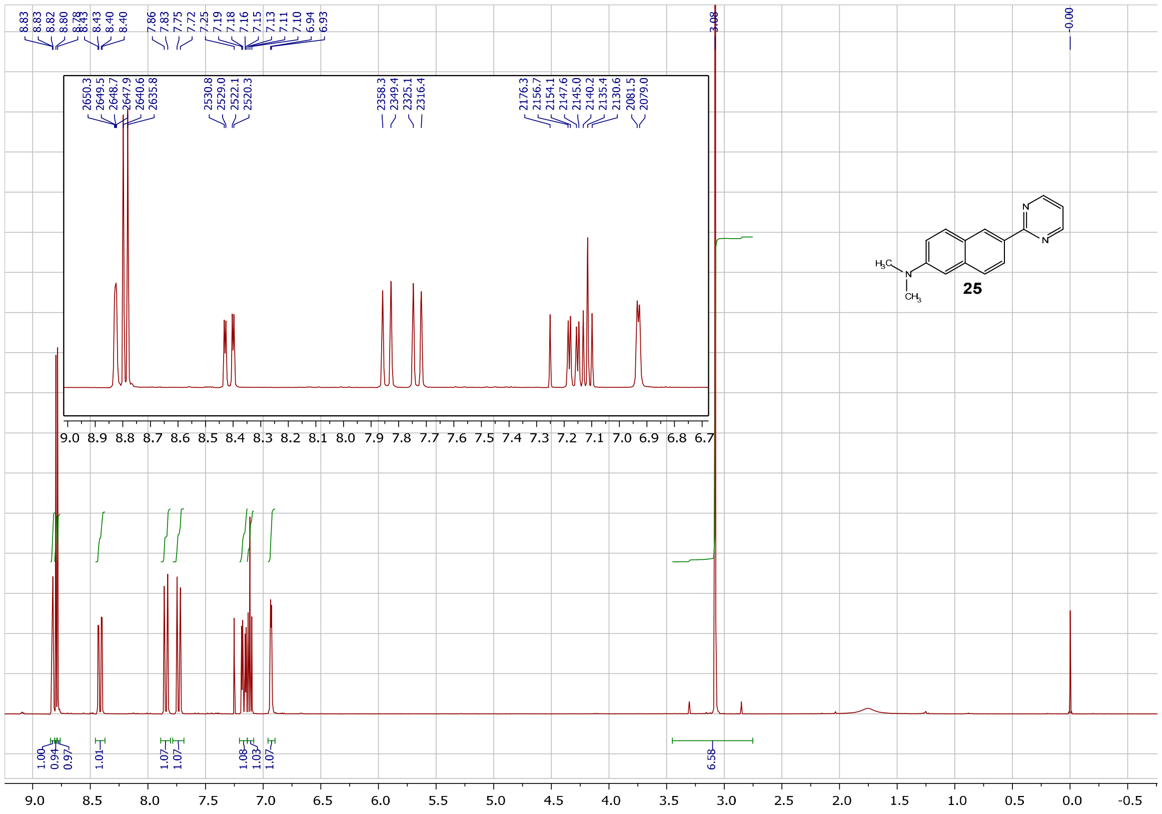

Figure S38. ^{1}H-NMR spectrum of the compound **25**.

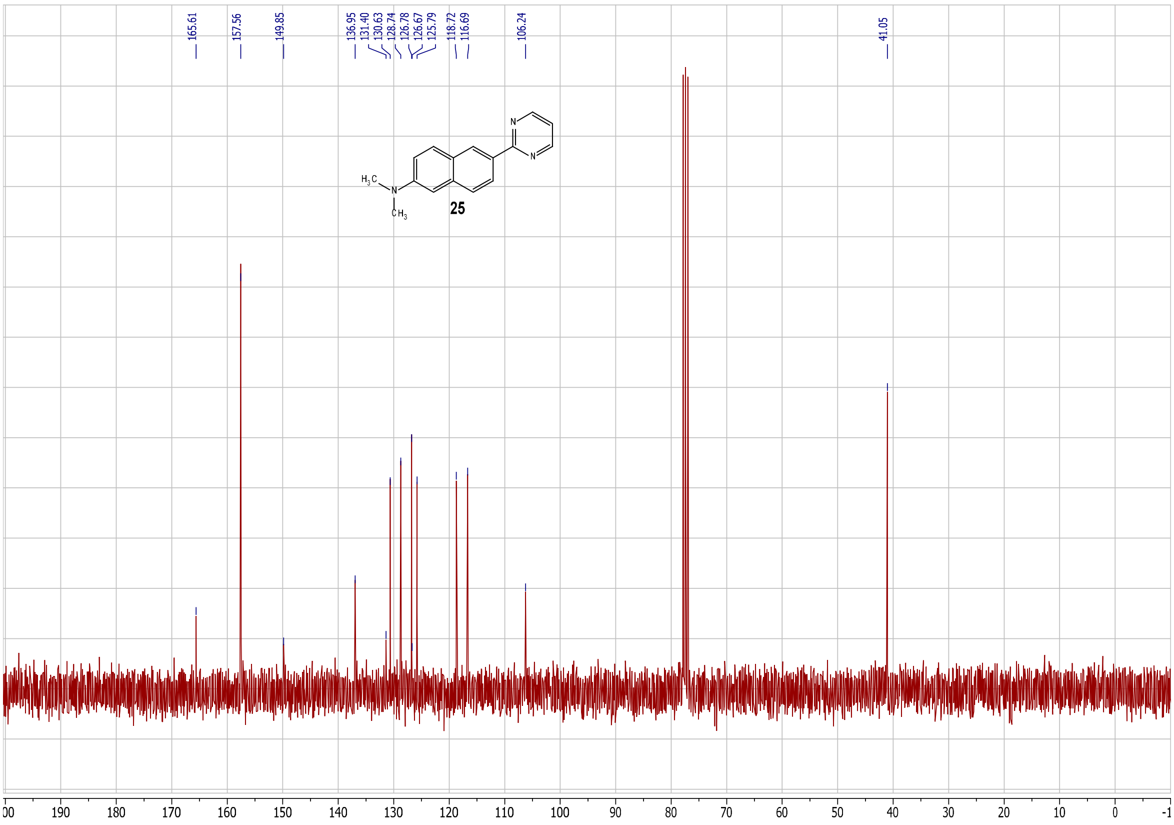

Figure S39. ^{13}C-NMR spectrum of the compound **25**.

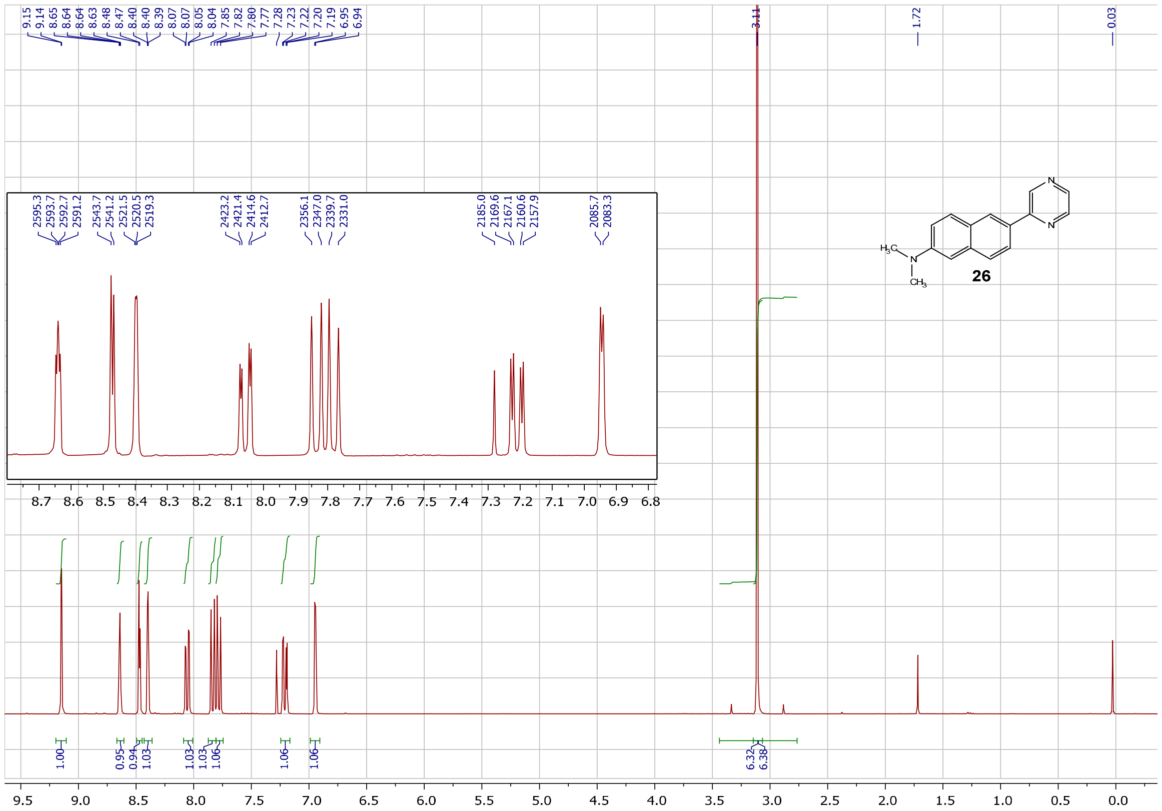

Figure S40. ^{1}H-NMR spectrum of the compound **26**.

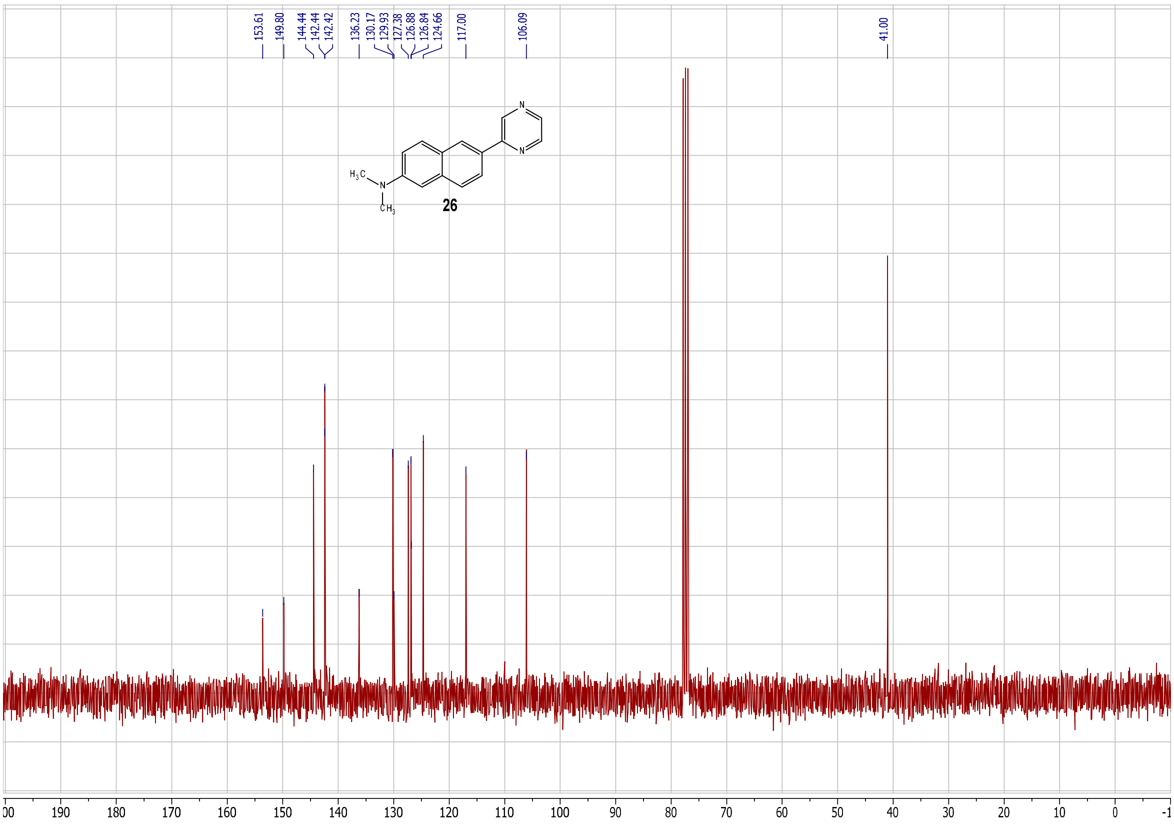

Figure S41. ^{13}C-NMR spectrum of the compound **26**.

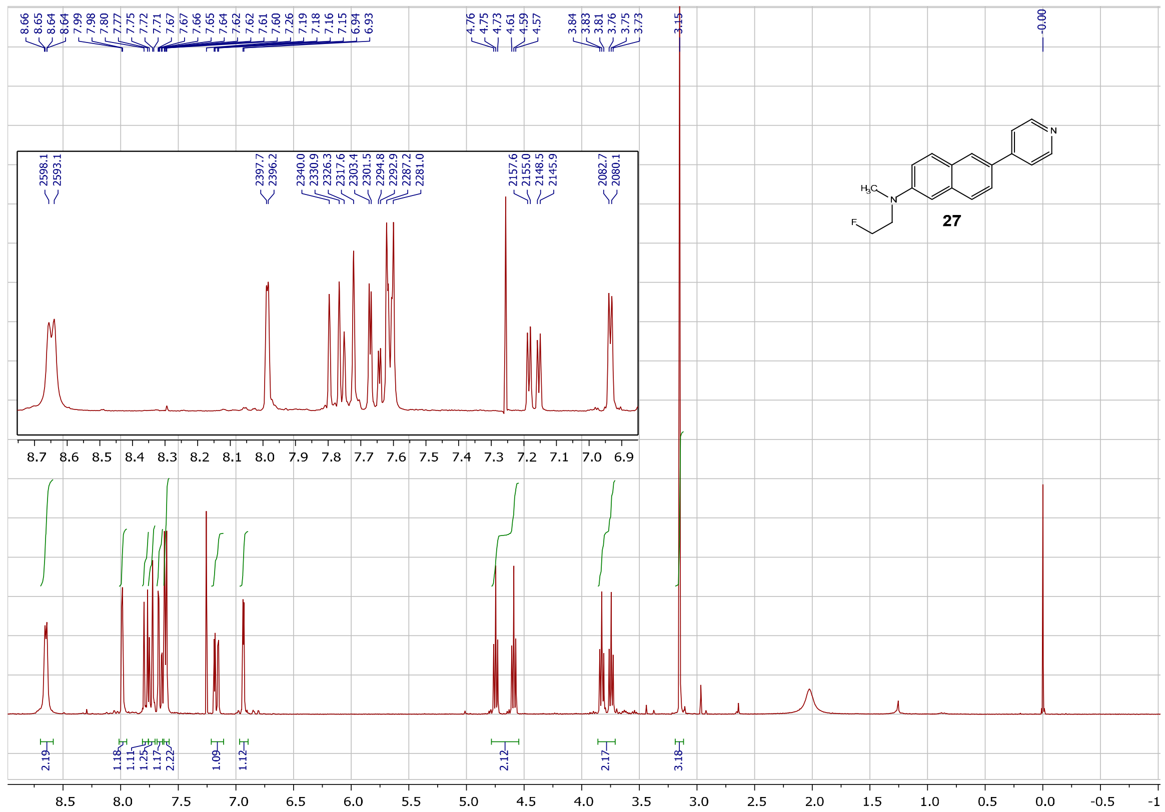

Figure S42. ^{1}H-NMR spectrum of the compound **27**.

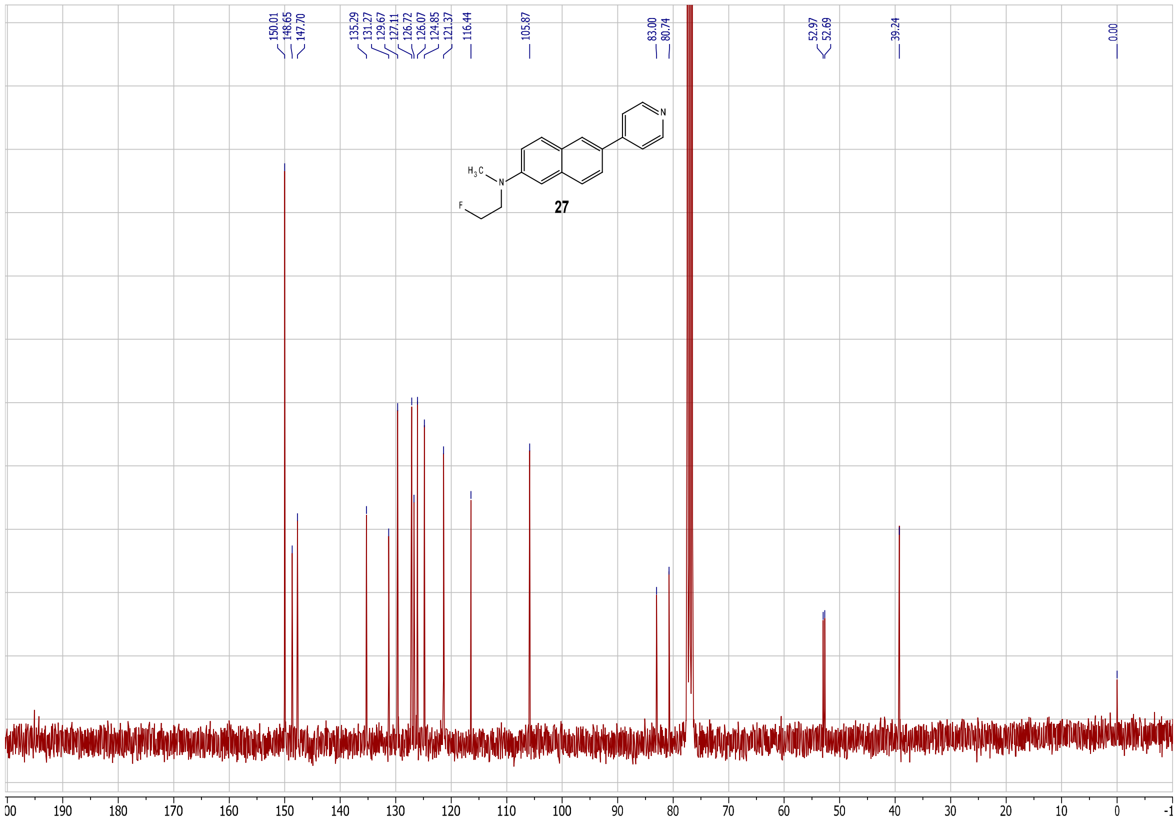

Figure S43. ^{13}C-NMR spectrum of the compound **27**.
